# Hepatorenal protective effect of berberine-loaded Ag–ZnO nanoparticles in mitigating carbon tetrachloride (direct) and alloxan-induced oxidative stress (indirect) in albino rat models

**DOI:** 10.1039/d6ra05117k

**Published:** 2026-09-22

**Authors:** Javeria Shafique, Aleena Ali, Manal Nazim, Mubashra Inam, Saed A. Althobaiti, Khalid S. Alotaibi, Mohamed Mohamed Soliman, Sumaira Anjum

**Affiliations:** a Department of Biotechnology, Kinnaird College for Women 93-Jail Road Lahore 54000 Pakistan javeriashafique710@gmail.com aleenaraza2002@gmail.com manalnazim123@gmail.com sumaira.anjum@kinnaird.edu.pk +92-3006957038; b Department of Medical Chemistry, Faculty of Medicine, University of Debrecen Egyetem Tér 1 4032 Debrecen Hungary bashrabutt@gmail.com; c Department of Biology, Turabah University College, Taif University P.O. Box 11099 Turabah Taif Saudi Arabia saed@tu.edu.sa; d General Science and English Language Department, College of Medical Sciences, AlMaarefa University Riyadh Saudi Arabia Kaotaibi@um.edu.sa; e Clinical Laboratory Sciences Department, Turabah University College, Taif University Taif-21995 Saudi Arabia mmsoliman@tu.edu.sa

## Abstract

Hepatorenal toxicity stems from chronic oxidative stress (OS) generated from mechanisms underlying chemical or metabolic injury. The present study investigates the hepatorenal toxicity induced by carbon tetrachloride (CCl_4_) and alloxan, and the hepatorenal-protective potential of Berberine-loaded (BBR) silver–zinc oxide nanoparticles (Ag–ZnO NPs) in mitigating the damage. The characterization of synthesized NPs was performed using UV-vis spectroscopy, XRD, FTIR, and TEM, DLS, zeta potential and TGA analysis. Synthesized NPs showed characteristic physicochemical properties in terms of size, shape and crystallinity. *In vivo* studies demonstrated that administration of CCl_4_ directly altered the levels of hepatic and renal function parameters, attributed to excessive generation of ROS compromising the antioxidant defense system, while alloxan directly targeted pancreatic β-cells, elevating blood glucose levels, generating ROS, which caused secondary hepatic and renal damage pertaining to persistent hyperglycemia. The lipid profile was also disrupted in both models, with increased levels of total cholesterol (TC), triglycerides (TG), and low-density lipoprotein (LDL), while reducing high-density lipoprotein (HDL) levels. Histopathological analysis also revealed significant changes in liver and kidney structure. BBR-loaded Ag–ZnO NPs were found to restore the hepatic and renal function parameters better in the CCl_4_ induced toxicity model, owing to their anti-oxidant and anti-inflammatory activity. Serum levels of TC, TG, and LDL were reduced while HDL levels were increased. Furthermore, the endogenous antioxidant defense system was restored to a greater extent. The structural integrity of liver and kidneys was also restored to normal. To conclude, the BBR-loaded Ag–ZnO NPs exhibited an exceptional potential in overcoming oxidative stress-induced direct toxicity by CCl_4_, as compared to indirect (metabolic) toxicity induced by alloxan.

## Abstract 2

Hepatorenal toxicity stems from chronic oxidative stress (OS) generated from mechanisms underlying chemical or metabolic injury.

## Graphical abstract

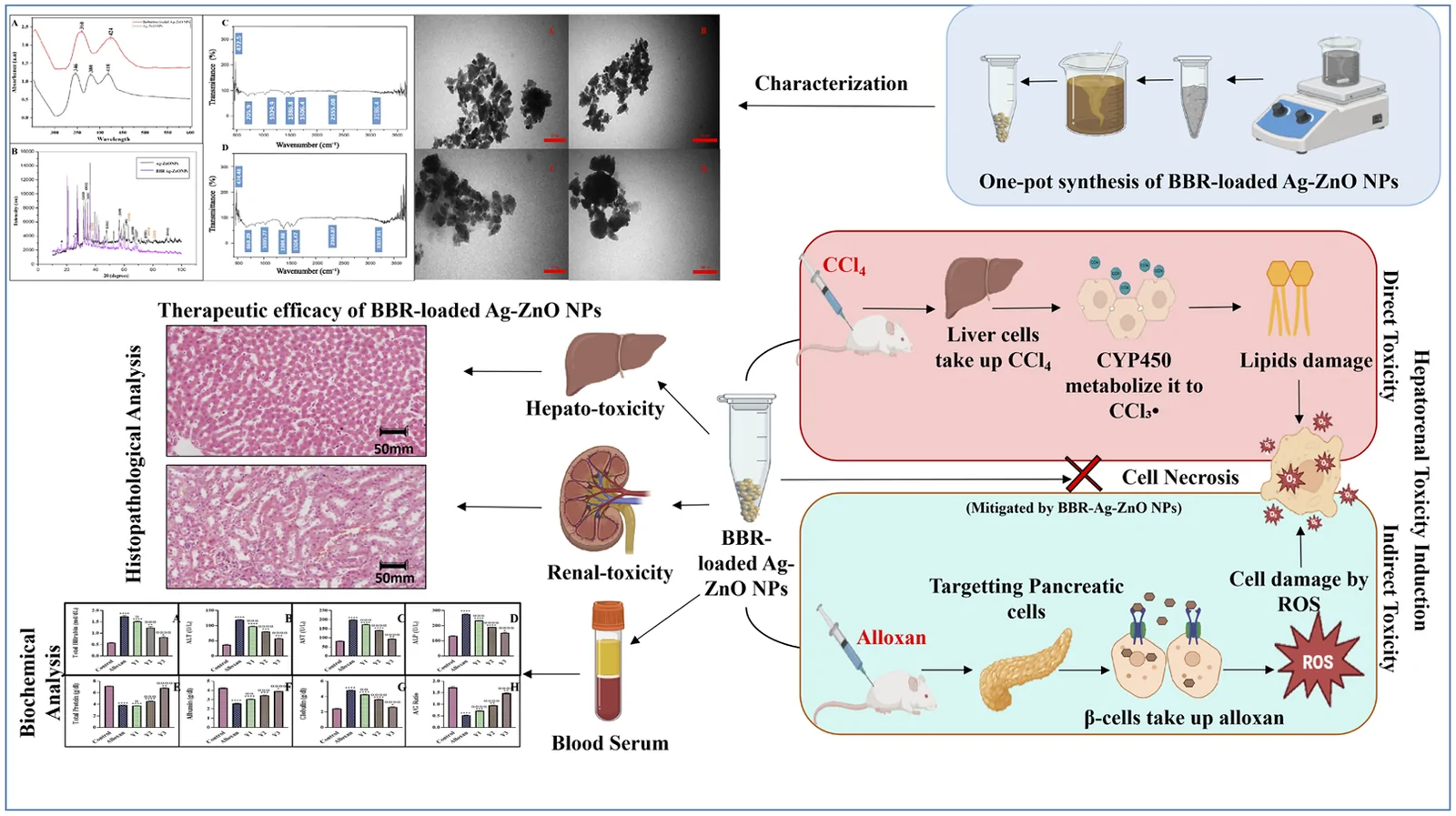

## Introduction

Hepatorenal toxicity is defined as the simultaneous damage or dysfunction of the liver and the kidneys.^1,2^ One of the major underlying factors that contribute to hepatorenal damage is oxidative stress (OS), which serves by elevating the production of Reactive Oxygen Species (ROS) and Reactive Nitrogen Species (RNS), which surpasses the antioxidant defense capacity of the body, consequently initiating a chain of molecular events that undermine both hepatic and renal structure and function.^3–6^ The primary contributors of elevated ROS in liver are, but not limited to, activated Kupffer cells, NADPH oxidases, and mitochondrial dysfunction, which synergistically initiate lipid peroxidation, apoptosis of hepatocyte, activation of stellate-cell, and onset of fibrosis, and if left untreated for long-term, can lead to cirrhosis and hepatocellular carcinoma.^3,7^ Likewise in the renal tissues, the dysfunctional mitochondria, lipid peroxidation, and protein oxidation are attributed to oxidative stress, which further leads to severe inflammation of interstitial tissues and tubular damage, diminished renal capacity of glomerular filtration, and depletion of antioxidants.^8,9^ Furthermore, endotoxemia, portal hypertension, and systematic inflammation are some mechanisms that are triggered by advanced liver diseases, contributing to oxidative stress, and creating a cycle that worsens the renal failure and facilitates hepatorenal syndrome.^6,10^ Because the mechanisms linked with oxidative stress are both causative and amplifying, there is a dire need to reduce the oxidative burden before the onset of serious complications.

Two of the widely utilized hepatorenal toxicants are carbon tetrachloride (CCl_4_) and alloxan monohydrate.^11–13^ Alloxan is widely used to induce experimental diabetes through ROS-mediated β-cell destruction, that results in oxidative injury to liver and kidney tissues.^14,15^ Whereas carbon tetrachloride (CCl_4_) undergoes bioactivation by hepatic cyctochrome P450 to form the reactive 
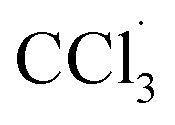
 (trichloromethyl) and CCl_3_OO˙ (trichloromethyl peroxy) free radicals which disrupt membrane integrity, initiates lipid peroxidation and causes inflammation which results in histopathological alterations and elevated liver function enzymes.^16,17^

The conventional approaches for the management of hepatorenal damage include anti-inflammatory drugs, antioxidants, and supportive therapies.^18,19^ For example, to treat an overdose of acetaminophen, *N*-acetylcysteine is used.^20,21^ Similarly for chronic liver inflammation and kidney damage, conventionally, corticosteroids and angiotensin-converting enzyme inhibitors and diuretics are used, respectively.^22^ Nevertheless, conventional therapies have limitations such as organ cross-toxicity, long-term complications, and side-effects. These therapies are effective in specific clinical settings, however they mainly alleviate disease manifestation but don't promote tissue healing or deal with underlying damage.^23^ In areas with limited resources, like Pakistan, the accessibility along with medication cost, may pose major challenges to the management of hepatorenal disorders.

Many of the plants containing high levels of antioxidants like phenols, flavonoids, tannins, alkaloids, and vitamins have been found helpful against hepatorenal toxicity, owing to their ability to neutralize reactive oxygen species, suppressing lipid peroxidation, and enhancing the antioxidant defenses in both liver and kidney tissues.^24–26^ Berberine (BBR), an alkaloid, produced by some plants, specifically species of *Berberis*, has demonstrated dual hepato- and renal-protective potential in experimental models by mitigating OS and inflammation *via* activation of Nrf2 signaling pathway and suppression of NF-κB signaling, improving endogenous antioxidant defense, reducing tissue damage, and restoring biochemical markers.^27–34^

Despite that, on account of first-pass effect and poor bioavailability, solubility, and a rapid metabolism, the clinical application of BBR is limited.^27,28^

The encapsulation of bioactive compounds into nanoparticles can improve the solubility, increase cellular absorption, and targets the damaged liver and kidney tissues alongside reducing the off-target effects.^29–31^ Among the nanoparticles (NPs), silver (Ag) and zinc oxide (ZnO) nanoparticles have been reported to exhibit hepatorenal protective potential by modulating key oxidative stress pathways in both hepatic and renal tissues.^32–34^ Ag NPs have been reported to enhance the antioxidant enzymes like CAT and SOD, up-regulate the antioxidant defense system, but at high dose Ag NPs may also contribute to the production of ROS through the release of Ag^+^, disrupting the mitochondrial.^35,36^ Similarly, ZnO NPs have been shown to restore the antioxidant enzymes SOD and CAT by activating NRf2, and have reduced lipid peroxidation in hepatorenal toxicity models.^37,38^ In addition to that, ZnO NPs have been reported to mitigate the expression of pro-inflammatory cytokines like tumor necrosis factor (TNF), by down-regulating NF-κB, the key inflammatory pathway in damaged tissues.^37,39^ However, under severe OS, ZnO NPs have been shown to down-regulate the expression of antioxidant gene, consequently exacerbating OS.^40^

Various studies have reported that the antioxidant and anti-inflammatory properties exhibited by Ag–ZnO bimetallic nanoparticles are higher than mono-metallic nanoparticles.^41,42^ Thus for the encapsulation of BBR, Ag–ZnO bimetallic NPs would be the ideal candidate owing to the exceptional synergistic antimicrobial, cytoprotective, anti-inflammatory, and anti-oxidant activities.^43,44^ Therefore, this research aims to compare the therapeutic efficacy of BBR-loaded Ag–ZnO bimetallic nanoparticles against CCl_4_-induced and alloxan-induced hepatorenal toxicity, for enhanced anti-inflammatory and anti-oxidant activity, and hepatorenal biocompatibility in albino rats.

## Materials and methods

### Chemicals

Silver nitrate (AgNO_3_), zinc acetate dihydrate (Zn(CH_3_COO)_2_·2H_2_O), sodium hydroxide (NaOH), berberine chloride (BBR), carbon tetrachloride (CCl_4_), alloxan monohydrate, 2,2 diphenyl-1-picrylhydrzyl (DPPH) were purchased from Sigma-Aldrich. All other chemicals and reagents obtained were of analytical grade and were used without further purification.

### Methodology

#### Synthesis of Ag–ZnO bimetallic nanoparticles

Ag–ZnO bimetallic NPs were synthesized *via* single–pot reaction using chemical co-precipitation method. Silver nitrate and zinc acetate dihydrate were used as precursor salts. A 0.1 M solution was used based on literature reporting high yield.^45^ A 0.1 M solution of zinc acetate dihydrate was prepared by dissolving 4.38 g in 200 mL distilled water, while 0.1 M solution of silver nitrate was prepared by dissolving 1.69 g of silver nitrate in 100 mL of distilled water, followed by mixing of both solutions on magnetic stirrer. During stirring, sodium hydroxide (NaOH) was added drop-wise as a precipitating agent, until the pH reached 12. The mixture was then stirred further for 2 h to ensure reaction completion. The formation of light brown or greenish-gray precipitates ensured the synthesis of Ag–ZnO nanoparticles. The solution was then centrifuged at 12 000 rotations per minute (rpm) for 15 minutes to get the pallets of Ag–ZnO NPs, discarding the supernatant. After collection of pallets, they were washed thrice before drying them at 37 °C for 2 days to obtain pure Ag–ZnO bimetallic nanoparticles.^42^

#### Preparation of berberine (BBR) loaded Ag–ZnO bimetallic nanoparticles

A solution of BBR was prepared at a concentration of 2.5 mg mL^−1^ in ethanol, and loaded onto Ag–ZnO NPs at an equal ratio of 1 : 1, under continuous stirring at 40 °C for about 3 hours. The pH was maintained at 7.4, followed by centrifugation of the solution at 12 000 rpm for 15 minutes to get the pallets, the supernatant obtained from this step was stored for calculation of encapsulation efficiency. The pallets were then dried at 25 °C for 24 hours, and resultant BBR-loaded Ag–ZnO NPs were stored for further analysis.^46^

#### Encapsulation efficiency estimation

The encapsulation efficiency was determined by analyzing supernatant containing free amount of BBR. Briefly, a calibration curve of BBR was plotted using a series of standard concentrations (0, 10, 25, 50, 75, 100, 125 µL) and their absorbance was measured utilizing UV-visible spectrophotometry. The calibration curve was evaluated for linearity, and the regression equation obtained from it (*y* = 0.0117*x* + 0.05) was used to calculate the concentration of free BBR in the supernatant. The calibration curve demonstrated good linearity (*R*^2^ = 0.9938). The measured concentration and supernatant volume were then used to calculate the amount of free BBR. Encapsulation efficiency was subsequently calculated using encapsulation efficiency formula;^47^
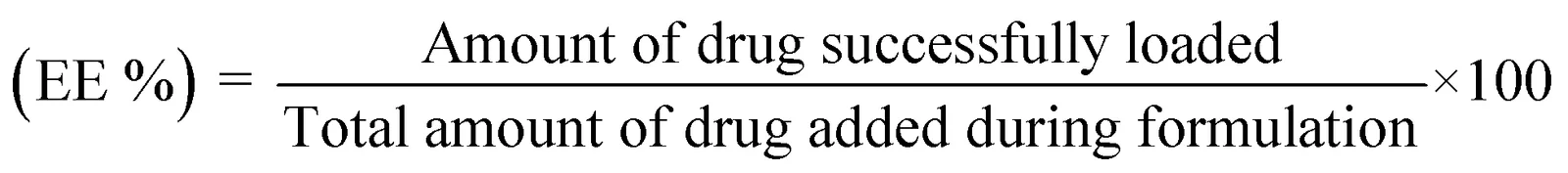


#### Physio-chemical characterization of Ag–ZnO nanoparticles and BBR-loaded Ag–ZnO nanoparticles

Various characterization techniques were employed to confirm the morphology and physio-chemical properties of the synthesized Ag–ZnO NPs and BBR-loaded Ag–ZnO NPs. To confirm the synthesis and encapsulation efficiency of Ag–ZnO NPs, UV-visible spectrophotometer (Thermo Scientific SPECTRONIC 200) was utilized, where peaks were observed within the wavelength range of 300 to 600 nm. Fourier Infrared Transform (FTIR) spectra (FTIR Thermo Scientific Nicolet iS50) were recorded between 400–4000 cm^−1^, to analyze functional groups and chemical interactions between the NPs and BBR. The crystalline nature of the nanoparticles was analyzed using X-ray diffraction (Thermo Scientific ARL EQUINOX 100). The diffraction patterns were recorded at 2*θ* range of 20–80° using CuKα radiation. The size of the Ag–ZnO and BBR-loaded Ag–ZnO nanoparticles was also calculated using Debye–Scherrer equation:^48^
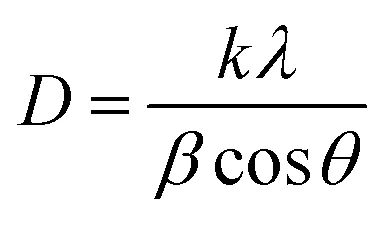
where, *k* = shape factor (0.9); *λ* = X-ray wavelength (1.5418 Å for CuKα radiation), *β* = full width at half maximum- FWHM (radians); *θ* = Bragg's angle (radians).

Dynamic light scattering (DLS) was employed to determine the hydrodynamic diameter and polydispersity index (PDI) of synthesized NPs, while the colloidal stability along with surface charge was measured through zeta potential utilizing the Zeta Potential Analyzer (90 Plus Particle Size Analyzer with Zeta Plus Software, Brookhaven Instruments Corporation, US). Transmission Electron Microscopy (TEM) (Thermo Scientific Spectra 300) was employed to further confirm the morphology and size of the synthesized nano-formulations. The TEM measurements were performed on multiple independent batches to ensure the uniformity in terms of size and shapes of NPs. ImageJ software was further utilized to determine the particles size from TEM micro-graphs. Using a UV-visible spectrophotometer, the absorption profiles of both Ag–ZnO and BBR-loaded Ag–ZnO NPs were monitored at predetermined storage intervals to assess their stability. The thermal stability of both NPs was evaluated through thermogravimetric analysis (TGA) using Mettler Toledo™ TGA/DSC 3+ and the change in weight of samples was recorded as function of increasing temperature.

#### Biocompatibility analysis of Ag–ZnO NPs and BBR-loaded Ag–ZnO NPs

To analyze the biocompatibility of both NPs, brine shrimp lethality assay and hemo-compatibility analysis on RBCs was performed. A concentration of 25–400 µg mL^−1^ and 5–200 µg mL^−1^ of both formulations was used for hemolysis and brine shrimp lethality assay, respectively. All the experiments were carried out in triplicates following the protocols reported by Inam *et al.*^49^ Formal consent was taken from the healthy participants, and the experiments were approved by the Ethical Review Committee of Kinnaird College for Women University, Lahore (DR-204). The human-subject component of the study was conducted in accordance with the ethical principles of the Declaration of Helsinki (1964) and its subsequent amendments were duly considered.

#### *In vivo* study on animal models

##### Animal maintenance

54 female albino rats weighing between 140–170 g were purchased from Institute of Biochemistry and Biotechnology, University of Veterinary and Animal Sciences, Lahore. The rats were kept in polypropylene cages and were given one week before initiating the experiment, to acclimatize and observe them to ensure the rats were healthy. The temperature of the room was set to 25 °C and the humidity level was 60%. A 12-hour light cycle was maintained for the rats and throughout the period food and water were given *ad libitum*. Ethical approval was provided by the Ethical Review Committee, Kinnaird College for Women University, Lahore (DR-204). This study was conducted in accordance with the ARRIVE guidelines. All the recommendations for the handling of lab animals given by National Health Institute (NIH) were followed (NIH Publication Number 85-23, Revised 1985). We have added details on the study design, animal care, sample size calculation and statistical analysis in accordance with these guidelines.

##### Experimental design

Two models were designed for toxicity study, employing two known toxicity agents, CCl_4_ and alloxan monohydrate. Both models had four groups each (*n* = 6 rats per group), with a common negative control. In both models, the doses given were reported as “Gold Standard” for inducing hepatorenal toxicity.^50–53^ In CCl_4_-induced toxicity model, each rat received intraperitoneal (IP) injection of CCl4 (1 ml Kg^−1^ BW) diluted with olive oil (1 : 1), twice a week for 3 weeks along with treatments (Table 1). Similarly, in alloxan-induced toxicity model, each rat received a single dose of 165 mg kg^−1^ BW alloxan *via* IP route.

**Table 1:** Summary of *in vivo* experimental groups and treatments received by albino rats

Groups	Treatment received
Group 1 (negative control)	Rats received normal diet and saline water for 21 days

**CCl**_**4**_ **-induced toxicity model**	
Group 2 (positive control)	Rats received CCl_4_ twice a week for 21 days
Group 3 (T1)	Rats received CCl_4_ twice a week for 21 days and BBR (150 mg Kg^−1^ BW) orally, daily for 21 days
Group 4 (T2)	Rats received CCl_4_ twice a week for 21 days and Ag–ZnO NPs (10 mg Kg^−1^ BW) orally, daily for 21 days
Group 5 (T3)	Rats received CCl_4_ twice a week for 21 days and Ag–ZnO NPs (10 mg Kg^−1^ BW) orally, daily for 21 days

**Alloxan-induced toxicity model**	
Group 2 (positive control)	Rats received a single dose of alloxan to induce diabetes
Group 3 (Y1)	Rats received a single dose of alloxan, followed by a daily dose of BBR (150 mg Kg^−1^ BW) orally for 21 days upon confirmation of diabetes
Group 4 (Y2)	Rats received a single dose of alloxan, followed by a daily dose of Ag–ZnO NPs (10 mg Kg^−1^ BW) orally for 21 days upon confirmation of diabetes
Group 5 (Y3)	Rats received a single dose of alloxan, followed by a daily dose of BBR-loaded Ag–ZnO NPs (10 mg Kg^−1^ BW) orally for 21 days upon confirmation of diabetes

##### Confirmation of experimental diabetes

The Blood Glucose Levels (BGLs) of all alloxan-administered rats were measured before and after (72 h) the administration of alloxan to confirm the diabetic status of rats. Moreover, after the successful induction of diabetes, the BGLs were measured at regular intervals (Day 1, 7, 14, and 21) to keep track of diabetes.

##### Blood sample collection

Blood samples were collected at the end of the experiments for biochemical analysis after euthanizing all rats with 2.5% halothane. The samples were prepared prior to analysis. The serum was isolated, centrifuged at 3000 rpm for 5 min, transferred, and stored in clean test tubes.

##### Evaluation of hepatic function, renal function, and lipid profile

The levels of hepatic biomarkers including total bilirubin assay (TBL), alanine transaminase (ALT), aspartate transaminase (AST), alkaline phosphatase (APA), total protein (TP), albumin (ALB), and globulin (GB); renal biomarkers including creatinine and urea; and lipid profile activity including triglycerides (TG), total cholesterol (TC), high-density lipoproteins (HDL), and low-density lipoproteins (LDL) were measured using commercially available diagnostic kits, following the manufacturer's guidelines.

##### Evaluation of serum oxidative stress markers

To assess the oxidative stress in animal models, serum oxidative stress markers including malondialdehyde (MDA), reduced glutathione (GSH), superoxide dismutase (SOD), and catalase (CAT), were performed. Blood samples from each group were centrifuged at 3000 rpm for 5 minutes to separate the serum, which was further used for analysis. Commercially available kits (Thermo Fisher Scientific) were utilized to determine the activities of SOD and CAT, and levels of GSH and MDA, following the manufacturer's instructions.

##### Histopathological analysis

After animals were dissected, hepatic and renal tissues were excised and fixed immediately in 10% neutral-buffered formalin. Furthermore, the samples were dehydrated through different graded concentrations of ethanol, clarified in xylene, and embedded in paraffin. A rotatory microtome was employed to section the paraffin blocks into 4 µm thick slices. The sections were then stained with Hematoxylin and Eosin (H&E) for histological evaluation.^54^

##### Statistical analysis

Biochemical analyses were statistically evaluated using GraphPad Prism 11.0. The data including all values were expressed as mean ± SD. Since homogeneity of variance was not assumed, to assess the differences among groups, Brown–Forsythe and Welch's ANOVA were used followed by Dunnett's T3 multiple-comparisons test. And *p* ≤ 0.05 was considered statistically significant.

### Characterization of Ag–ZnO NPs and BBR–Ag–ZnO NPs

#### UV-visible spectrophotometry

**Fig. 1:**
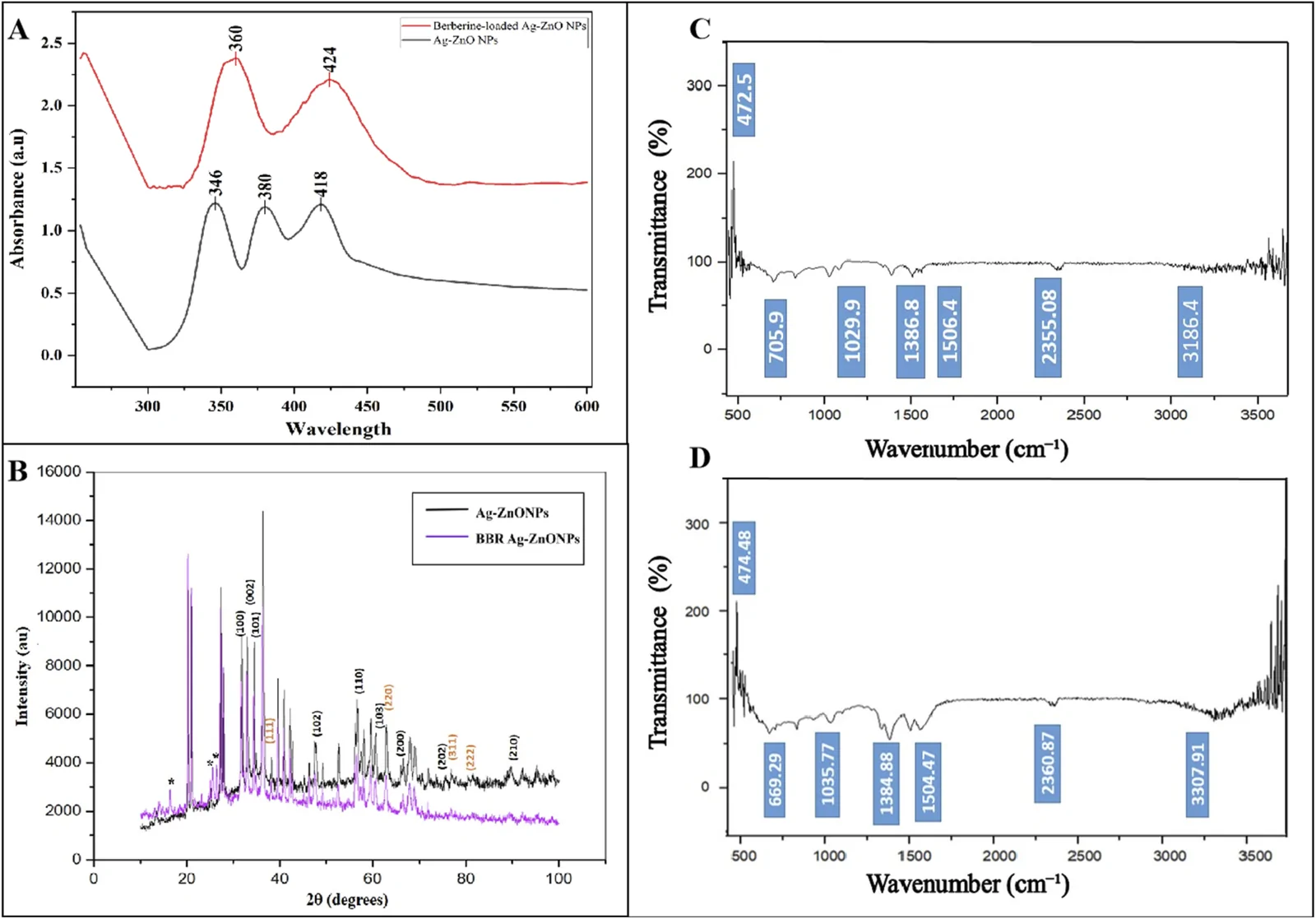
Physiochemical characterization of Ag–ZnO NPs and BBR-loaded Ag–ZnO NPs. (A) UV-visible spectrophotometry carried out between 300–600 nm (B) X-ray diffraction analysis, where black peaks correspond to Ag–ZnO NPs, purple peaks correspond to BBR-loaded Ag–ZnO NPs, and purple stars correspond to weak peaks of BBR; (C and D) Fourier transform infrared analysis of Ag–ZnO NPs (top) and BBR-loaded Ag–ZnO NPs (bottom) to determine the functional groups present in sample.

The synthesis of Ag–ZnO NPs and BBR-loaded Ag–ZnO NPs was confirmed using UV-visible spectra between the wavelength range of 300 to 600 nm. The synthesized Ag–ZnO NPs showed three distinctive absorption peaks at 346, 380, and 418 nm (Fig. 1A), corresponding to excitonic peaks of ZnO NPs and Surface Plasmon Resonance (SPR) peaks of Ag NPs.^55,56^ The observed absorption peaks are consistent with previous studies reporting absorption peaks of ZnO NPs in UV region (330–380 nm) and SPR band of Ag–ZnO NPs in visible region (400–450 nm).^40,57–59^ Additionally, the slight shift in ZnO peaks is attributed to synthesis temperature and size of particles.^60,61^

After the loading of berberine onto Ag–ZnO NPs, two distinctive peaks were shown by UV-vis spectrum at 360 nm and 424 nm, attributed to the plasmonic modification of Ag–ZnO NPs.^62^ This noticeable red-shift indicates non-covalent binding and surface complexation between berberine and Ag–ZnO NPs.^46,63^ Similar patterns of spectral changes have been reported for berberine interacting with ZnO NPs and other nano-carriers, demonstrating a shift of characteristic absorption bands upon drug loading, further supporting interaction between nano-carriers and berberine.^63–65^

#### X-ray diffraction analysis

The crystalline nature of synthesized Ag–ZnO NPs was further confirmed by XRD analysis. The XRD analysis revealed distinct peaks at 2*θ* values of 31.77° (100), 34.5° (002), 36.32° (101), 47.54° (102), 56.60° (110), 62.89° (103), 66.46° (200), 67.96° (112), 68.96° (201), 76.82° (202), 89.61° (203), 92.19° (210), 95.18° (211), and 98.67° (114), which corresponds to the hexagonal wurtzite structure of ZnO NPs in alignment with JCPDS #36-1451.^46,55^ Similarly, diffraction peaks at 38.1° (111), 64.42° (220), 77.43° (311), 81.47° (222), and 97.90° (400) indicate the face-centered cubic (FCC) structure of silver nanoparticles in correspondence to JCPDS #04-0783.^46^ The peaks corresponding to Ag–ZnO NPs are shown in Fig. 1B.

After loading of berberine onto Ag–ZnO NPs, the pattern demonstrated a slight shift in existing peaks along with the emergence of new peaks at 16.38°, 25.54°, and 26.32. Esnaashari *et al.*, reported a similar pattern of XRD for BBR–ZnO NPs, where diffraction peaks observed at 2*θ* angles of 16.32°, 24.54°, and 25.56° were attributed to berberine.^66^ Though these peaks were less distinct and sharp in comparison to the peaks exhibited by Ag–ZnO NPs, they are still consistent with previously reported nano-composite systems, where BBR and other drug molecules either transitioned to less crystalline form or emerged as weak peaks in XRD patterns, following loading onto nanoparticles, confirming the strong interaction of BBR with Ag–ZnO NPs in this study.^66–69^ Moreover, the diffraction peaks observed at lower angle (2*θ* ≈ 21–25°) correspond to secondary crystalline phase, likely Zn(OH)_2_, attributed to partial dehydration during alkaline synthesis as reported in literature.^70–72^

The shifts observed in XRD pattern are directly related to morphology and size of NPs. Debye–Scherrer equation was employed to calculate the size of both Ag–ZnO NPs and BBR-loaded Ag–ZnO NPs, which was 19.291 nm and 22.659 nm, respectively. The results indicate a slight shift towards increased size, following BBR loading. In a study, Givarian *et al.*, reported that increasing the concentration of berberine chloride from 4 mg to 7 mg in gelatin/perfluorohexane (PFH) nanodroplets significantly increased the size of NPs from 278.2 nm to 351.2 nm, revealing a direct relation between quantity of drug and size of NPs.^73^ Various nano-carriers have demonstrated similar effects following drug encapsulation, where the increased size of nanoparticles is attributed to structural modifications induced by drug intercalation or surface coating.^47,63,74–76^

#### FTIR analysis

FTIR analysis was performed to analyze the functional groups present and surface interactions in both Ag–ZnO NPs and BBR-loaded Ag–ZnO NPs.^77^ The FTIR spectrum displayed characteristic peaks at 472.5 cm^−1^ and 705.9 cm^−1^ corresponding to the stretching vibrations of Zn–O, which is consistent with metal–oxygen bond formation.^78^ Additional peaks 1386 cm^−1^suggest the presence of COO^−^ groups, while the band at 1506.40 cm^−1^corresponds to the stretching of aromatic C=C.^79–82^ The broad absorption around 3186.40 cm^−1^ corresponds to OH stretching originating from the hydroxyl groups as shown in Fig. 1C.^82^

FTIR analysis of BBR-loaded Ag–ZnO NPs revealed profound spectral shifts as well as characteristic peaks followed by BBR incorporation into Ag–ZnO NPs (Fig. 1D). The shift in characteristic FTIR band is attributed to surface interactions and functional group modifications upon drug loading.^83^ The Zn–O stretching peaks shifted from 472.5 cm^−1^ and 705.9 cm^−1^ to 474.48 cm^−1^ and 669.29 cm^−1^, while peak corresponding to Zn–OH shifted from 1029.98 cm^−1^ to 1035.77 cm^−1^, suggesting modifications in surface bonding attributed to the binding of BBR to NPs, which is consistent with drug-metal oxide NPs.^84^ The emergence of new peaks at 536 cm^−1^, 1045 cm^−1^, 1568.12 cm^−1^, and 932 cm^−1^ suggest their correspondence to berberine.^43^

The minor shift of aromatic C=C band from 1506.40 cm^−1^ to 1504 cm^−1^ indicates non-covalent interactions between the aromatic rings of berberine and surface of Ag–ZnO NPs.^61^ Additionally, the O–H stretching band significantly shifted from 3186.40 cm^−1^ to 3307 cm^−1^, indicating the formation of hydrogen bond between between BBR and Ag–ZnO NPs. The same pattern of band shifting can be observed across multiple peaks, the peak shift from 1386 cm^−1^ to 1384.88 cm^−1^, the prominence attained by the peak shifting to1338 cm^−1^ from 1330 cm^−1^, and distinct shift of the ∼705 cm^−1^ region to 669.29 cm^−1^. Likewise, Nguyen *et al.*, studied the intramolecular interactions in BBR–ZnO and BBR–Ag NPs, and demonstrated significant shifts and changes in berberine's characteristic peaks in terms of position and intensity when combined with both ZnO and Ag NPs. In BBR–Ag, emergence of new peaks at 536 and 1045 cm^−1^ was observed, moreover, the peak of BBR at 731 cm^−1^ shifted to 727 cm^−1^, becoming more profound attributed to Ag NPs SPR behavior, which demonstrated a similar pattern observed in peak enhancement at 731 cm^−1^ in BBR-loaded Ag–ZnO NPs in the present study.^46^ This confirms the interactions and strong binding between BBR and Ag–ZnO NPs and its successful loading onto NPs.

#### Transmission electron microscopy

**Fig. 2:**
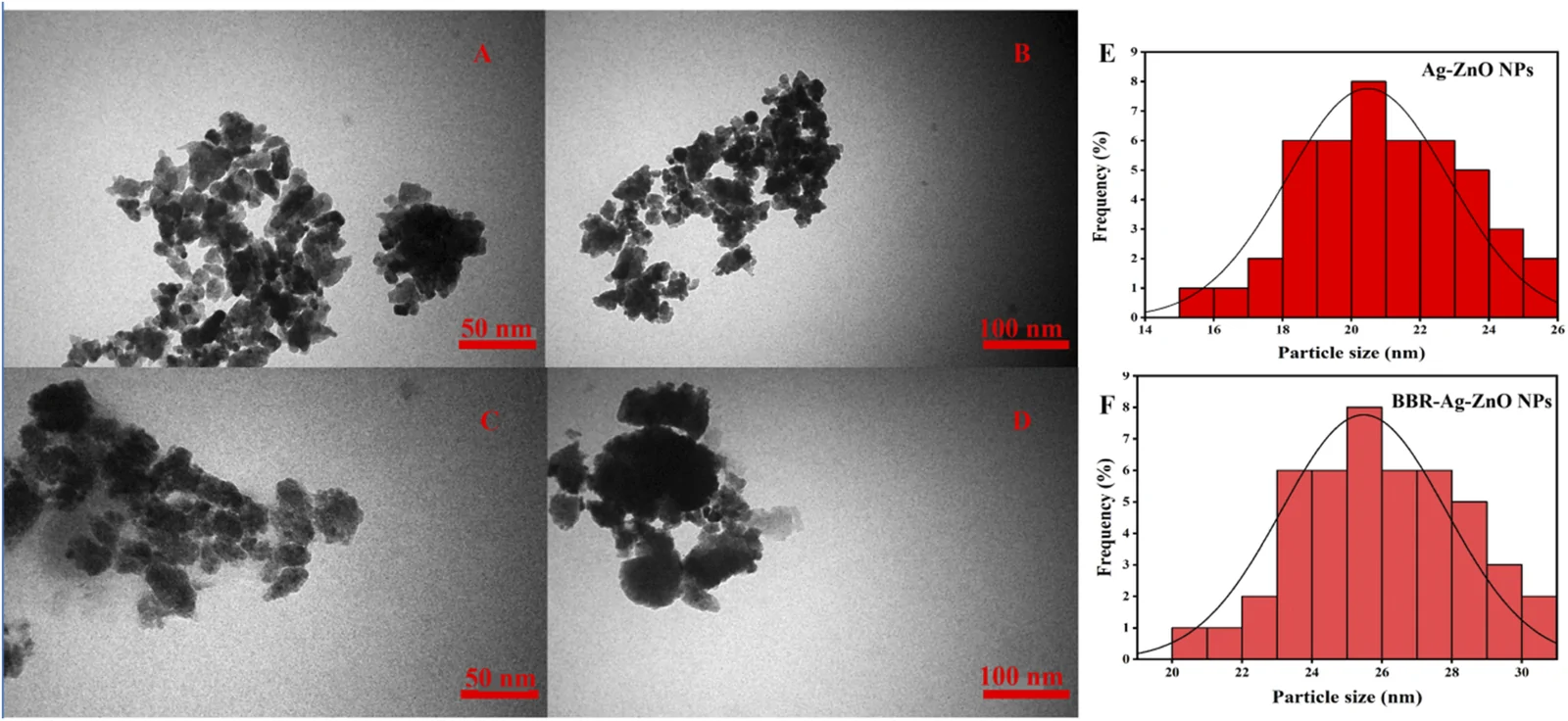
TEM analysis of (A) Ag–ZnO NPs (50 nm), (B) Ag–ZnO NPs (100 nm), (C) BBR-loaded Ag–ZnO NPs (50 nm), and (D) BBR-loaded Ag–ZnO NPs (100 nm). Particle size distribution histogram of (E) Ag–ZnO NPs demonstrating the size range from 15 to 26 nm and (F) BBR-loaded Ag–ZnO NPs demonstrating the size range from 20 to 32 nm.

TEM micrographs revealed dense and irregular clusters of well-defined primary particles in the synthesized Ag–ZnO NPs, exhibiting high electron contrast, typical of metal–oxides (Fig. 2A and B). Heterogeneous nucleation of Ag on ZnO surface was indicated by the quasi-spherical to slightly faceted morphology of the particles, which is characteristic for Ag–ZnO NPs synthesized *via* chemical precipitation or reduction method, and is consistent with previously reported morphology of Ag–ZnO NPs systems.^85–89^ In the micrograph, the crystalline base is formed by ZnO, while Ag contributes in enhancing the electron density and connectivity between particles.^90–92^ The observed agglomeration is attributed to the strong van der Waals interactions, high surface energy between NPs, their small size, and concentration of Ag, which causes aggregation.^93–96^ Employing the ImageJ software, the average particle size was calculated to be 22.42 nm, furthermore, the particle size distribution histogram was also generated (Fig. 2E). In contradiction to that, TEM micrographs of BBR-loaded Ag–ZnO NPs exhibited similar dense aggregates but they demonstrated larger and more irregular clusters (Fig. 2C and D), having an average size of 28.30 nm. The slight increase in the particles size aligns with the reported literature suggesting drug loading can cause surface coating, boundary enlargement of particles, and mild aggregation attributed to changes in surface charge.^47,97–99^ Furthermore, the sustained quasi-spherical morphology and high-electron density contrast indicates the stability of Ag–ZnO NPs after loading of BBR. Nguyen *et al.*, reported that BBR conjugation influenced the growth of both Ag/BBR and ZnO/BBR complexes, reporting that BBR tends to form small core/shell structures, without drastically altering the morphology or compromising the functionality of the nano-based delivery system.^46^ Likewise, it is reported that NPs within the range of 20–30 nm exhibit optimal biological activities, attributed to their enhanced membrane permeability and cellular uptake, and large surface to volume ratio, all of which contribute in enhancing the therapeutic efficacy.^100–102^

#### Evaluation of hydrodynamic diameter and colloidal stability of NPs

**Fig. 3:**
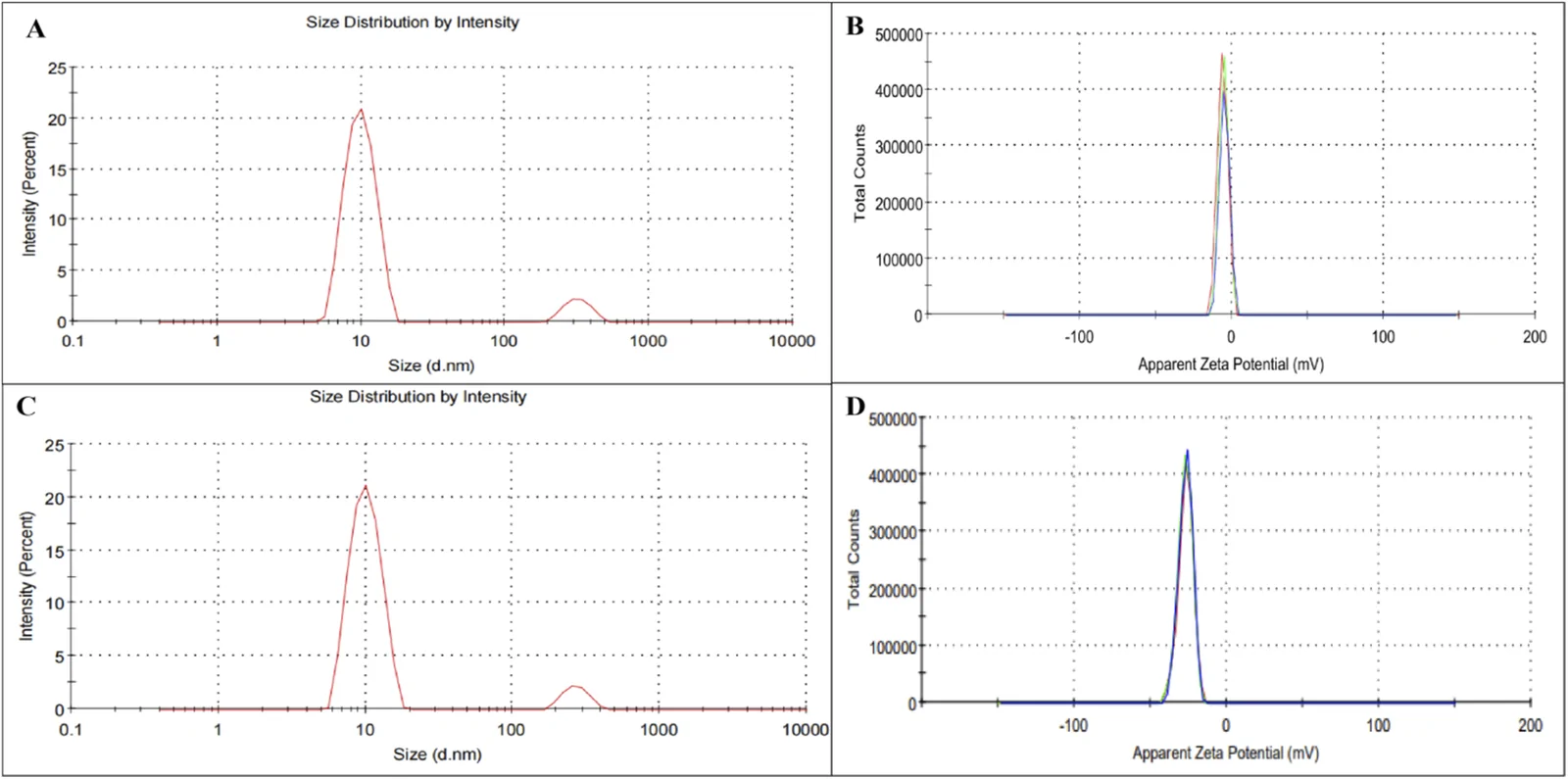
(A) Size distribution of Ag–ZnO NPs determined through DLS and (B) zeta potential of Ag–ZnO NPs. (C) Size distribution of BBR-loaded Ag–ZnO NPs determined through DLS and (D) zeta potential of BBR-loaded Ag–ZnO NPs.

To evaluate the hydrodynamic diameter and polydispersity index (PDI) of both Ag–ZnO NPs and BBR-loaded Ag–ZnO NPs, DLS was employed, while their colloidal stability was determined through zeta potential analysis (Fig. 3). The average hydrodynamic diameter of Ag–ZnO NPs and BBR-loaded Ag–ZnO NPs was 77.32 ± 2.31 nm and 86.08 ± 2.36 nm, respectively. The corresponding PDI values were 0.248 and 0.182 for Ag–ZnO NPs and BBR-loaded Ag–ZnO NPs, indicative of relatively narrow particle size distribution for both NPs formulations. The lower PDI observed for BBR-loaded Ag–ZnO NPs further suggests more homogeneous dispersion of NPs and narrow particle size distribution compared to Ag–ZnO NPs.

The increase in hydrodynamic diameter upon BBR loading suggests its adsorption onto the surface of NPs. Unlike TEM and XRD, which measure the physical diameter of dried NPs and average crystallite size, respectively, DLS determines the hydrodynamic diameter of NPs disperse in liquid medium, encompassing the NPs core along with surface coating, hydration layer, and electrical double layer. This corroborates our findings, where the particle size determined by TEM (22–28 nm) and crystallite size calculated by XRD (19–22 nm) exceeded the hydrodynamic diameter.^103–106^

The zeta potential of Ag–ZnO NPs and BBR-loaded Ag–ZnO NPs was determined to be −4.68 ± 1.14 mV and −26.9 ± 4.47 mV, respectively. This shift of zeta potential towards a more negative surface charge suggests stronger repulsion between the NPs following surface functionalization with BBR, indicating improved colloidal stability compared to Ag–ZnO NPs, which is consistent with reported literature.^107,108^ Although NPs with zeta potential values exceeding ±30 mV are generally considered highly stable, NPs with negative zeta potential between −22 to −27 mV have been reported to exhibit favorable biological activity, including enhanced antioxidant activity and hepatoprotective potential.^109–111^ Overall, the reduced PDI with improved surface charge suggests favorable dispersion characteristics of BBR-loaded Ag–ZnO NPs that may support their biological performance.

#### Evaluation of stability of NPs over time and their thermogravimetric analysis

**Fig. 4:**
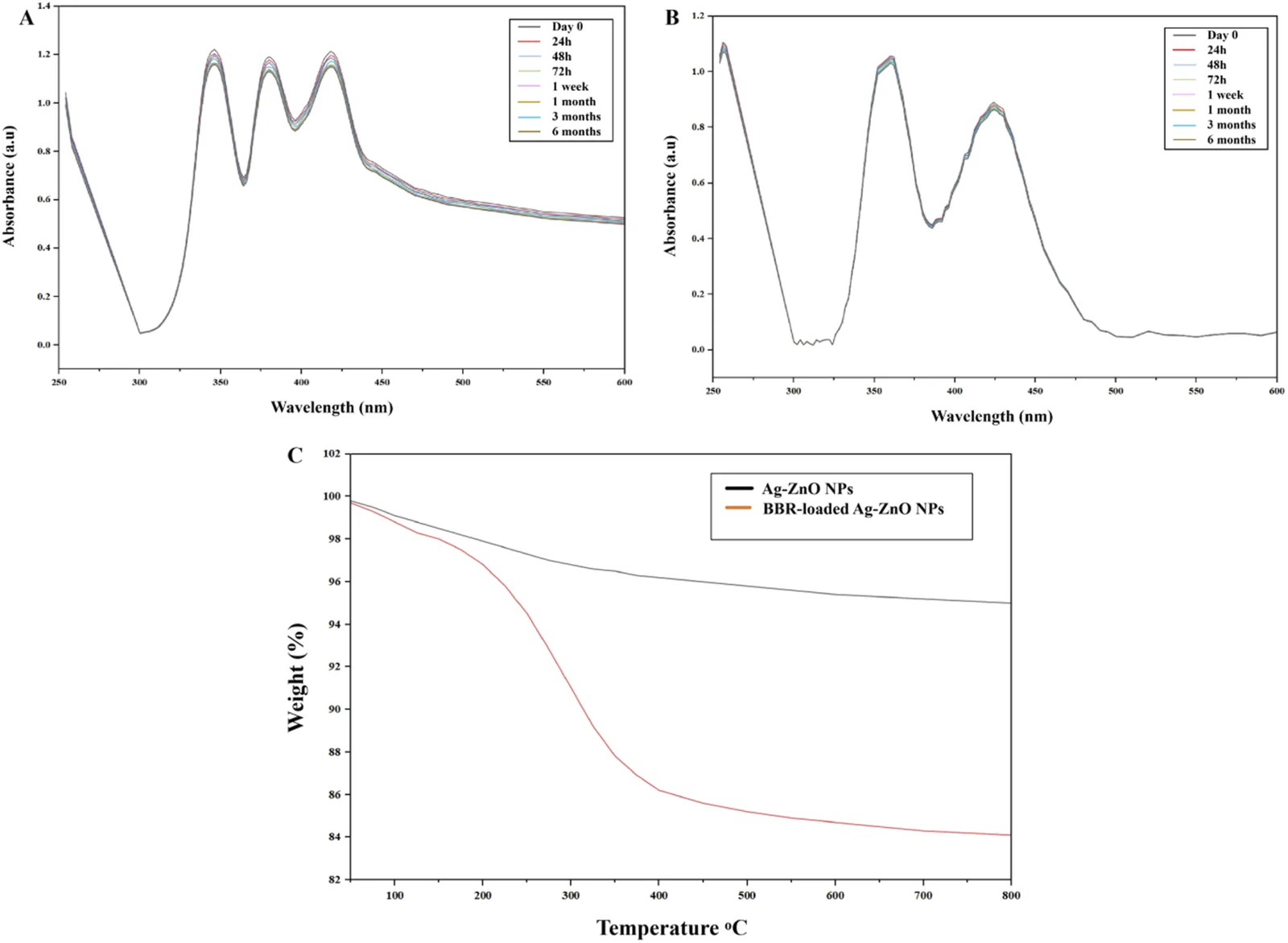
Evaluation of stability profile of (A) Ag–ZnO NPs and (B) BBR-loaded Ag–ZnO NPs over a span of 6 months and (C) TGA analysis of Ag–ZnO NPs (black) and BBR-loaded Ag–ZnO NPs (red) to assess their thermal stability.

The UV-vis absorption spectra of Ag–ZnO NPs and BBR-loaded Ag–ZnO NPs was observed over a period of six months to assess their stability (Fig. 4A and B). Throughout the study, no significant shift was observed in absorption spectra of both formulations, suggesting preservation of optical properties of NPs during this period. A slight decrease was observed in the absorbance intensity during the first week, after which the absorption spectra remained nearly unchanged suggesting the stability of NPs. Over six months, an overall decrease in absorbance of approximately 5% and 2.75% was exhibited by Ag–ZnO NPs and BBR-loaded Ag–ZnO NPs, respectively. The improved stability of BBR-loaded Ag–ZnO NPs is likely attributed to BBR coating which reduces the particle–particle interaction between NPs and subsequently limits aggregation. These observations corroborates studies demonstrating that surface functionalization of NPs improves their stability for prolonged periods while preserving their optical properties.^112–114^

To further evaluate the thermal stability of both nano-formulations, TGA was performed, where Ag–ZnO NPs exhibited approximately 5% weight loss over the temperature range of 50–800 °C. The weight loss was observed mainly between 50–400 °C as demonstrated by the TGA curves (Fig. 4C). The weight loss below 150 °C can be attributed primarily to removal of moisture and physically adsorbed water molecules, while the removal of residual surface species, chemically adsorbed water, and unreacted precursors may comprise the second stage of slow and relatively small weight loss between 150–400 °C.^115–117^ No pronounced weight loss was observed above 400 °C indicating the thermal stability of Ag–ZnO NPs. Contrary to that, BBR-loaded Ag–ZnO NPs exhibited approximately 15.9% weight loss. In addition to the initial moisture-associated weight loss, a pronounced loss was observed between 200–400 °C, which corroborates the thermal decomposition of surface bound organic coatings, supporting the successful coating of BBR onto Ag–ZnO NPs.^118^

### Biological compatibility and toxicity assessment of nanoparticles

#### RBC's hemolysis assay

The hemocompatibility of both Ag–ZnO NPs and BBR-loaded Ag–ZnO NPs was evaluated at different concentrations, with both formulations exhibiting a concentration-dependent increase in hemolysis. An increase in hemolysis from 1.45% to 3.93% for Ag–ZnO NPs was observed at 25 µg mL^−1^ and 400 µg mL^−1^, respectively. Whereas BBR-loaded Ag–ZnO NPs and free BBR exhibited values ranging from 0.92% to 2.62% and 0.76% to 2.21%, respectively, over the same range (Table 2). Similar dose-dependent results were reported by Salami *et al.*, and Onishchenko *et al.*, for ZnO NPs attributing it to ROS production and lipid peroxidation following an increase in concentration.^119,120^ Rahimimoghadam *et al.*, reported similar dose-dependent hemolytic activity of Ag NPs across concentrations of 1–1000 µg mL^−1^.^121^ Efthimiou *et al.*, reported that Ag NPs were more cytotoxic than ZnO NPs and Ag–ZnO NPs in mussel hemocytes across 0.1–50 µg mL^−1^, further suggesting that interaction between the NPs and their ionic counterparts could result in synergistic or antagonistic effects.^122^ Moreover, 3.16% ± 0.18% hemolysis by Ag–ZnO NPs has been reported in a previous study describing the NPs as slightly hemolytic.^123^ According to ASTM F756-based criteria, biomaterials with hemolysis under 5% are acceptable for human use, whereas the BBR-loaded Ag–ZnO NPs remained below 3% in this study suggesting their biocompatibility.^124^

**Table 2:** Hemolytic activity of free BBR, Ag–ZnO NPs, and BBR-loaded Ag–ZnO NPs at different concentrations

Concentration (µg mL^−1^)	Hemolysis (%)		
	Free BBR	Ag–ZnO NPs	BBR-loaded Ag–ZnO NPs
25	0.76 ± 0.12	1.45 ± 0.18	0.92 ± 0.14
50	1.08 ± 0.15	2.05 ± 0.21	1.31 ± 0.16
100	1.42 ± 0.18	2.58 ± 0.24	1.69 ± 0.19
200	1.76 ± 0.20	3.36 ± 0.28	2.05 ± 0.21
400	2.21 ± 0.22	3.93 ± 0.27	2.62 ± 0.24

#### Brine shrimp lethality assay

To assess the preliminary toxicity profile of the synthesized formulations, brine shrimp lethality assay was performed (Table 3). A concentration-dependent response was observed where mortality increased with increasing concentration of all three formulations. At 5 µg mL^−1^, mortality was 3 ± 0.1%, 12 ± 0.21%, and 7 ± 0.25% for BBR, Ag–ZnO NPs, and BBR-loaded Ag–ZnO NPs, respectively, which increased to 52 ± 0.19%, 99 ± 0.26%, and 86 ± 0.28%, respectively at 200 µg mL^−1^. The LC_50_ values were approximately 183.6 ± 0.19 µg mL^−1^, 23.2 ± 0.36 µg mL^−1^, and 42.6 ± 0.28 µg mL^−1^ for BBR, Ag–ZnO NPs, and BBR-loaded Ag–ZnO NPs, respectively. Anjum *et al.*, classified Ag–ZnO NPs as moderately toxic with LC_50_ value of 21.63 ± 0.61, which is comparable with our results.^125^ Abbasi *et al.*, reported LC_50_ values between 1–10 µg mL^−1^, 10–30 µg mL^−1^, and 30–100 µg mL^−1^ represent toxic compounds, moderately toxic compounds, and mildly toxic compounds, respectively.^126^ Among the nano-formulations, BBR-loaded Ag–ZnO NPs exhibited a higher LC_50_ value compared to Ag–ZnO NPs, indicating lower mortality response at equivalent concentrations, which may indicate that surface functionalization moderated the acute toxicity of Ag–ZnO NPs. However, brine shrimp assay does not directly predict toxicity or establish the mechanisms responsible for this difference.

**Table 3:** Concentration-dependent mortality of *Artemia salina* nauplii following exposure to free BBR, Ag–ZnO NPs, and BBR-loaded Ag–ZnO NPs

Concentration (µg mL^−1^)	Mortality (%)		
	Free BBR	Ag–ZnO NPs	BBR-loaded Ag–ZnO NPs
5	3 ± 0.1	12 ± 0.21	7 ± 0.25
10	7 ± 0.25	29 ± 0.14	17 ± 0.13
15	17 ± 0.13	41 ± 0.19	27 ± 0.11
20	21 ± 0.22	48 ± 0.15	36 ± 0.31
25	28 ± 0.17	54 ± 0.25	43 ± 0.15
50	35 ± 0.24	78 ± 0.28	57 ± 0.18
100	44 ± 0.27	93 ± 0.22	76 ± 0.21
200	52 ± 0.19	99 ± 0.26	86 ± 0.28

#### Encapsulation efficiency of berberine

Indirect quantification of free or non-encapsulated berberine left in the supernatant followed by centrifugation, was employed to calculate the encapsulation efficiency. Using the UV-vis spectrophotometer, a calibration curve of berberine was plotted between 340 to 350 nm. The values of supernatant absorbance were extrapolated onto the calibration curve to determine the concentration of free berberine. The value was subtracted from the total amount of berberine added per batch, during encapsulation. The resulting encapsulation efficiency was found to be 99.09%, which demonstrates the exceptionally high drug encapsulation capacity of Ag–ZnO NPs, suggesting strong affinity between BBR and Ag–ZnO NPs, attributed to interactions between the aromatic rings of berberine and hydroxyl groups of Ag–ZnO NPs.^46,127–129^

Akhtar *et al.*, reported the encapsulation efficiency of BBR into chitosan NPs to be around 91%, which is in accordance with our results.^67^ Dhivya *et al.*, employed PEG-PMMA copolymer modified ZnO NPs for the delivery of curcumin, reporting the loading efficiency of 92%, indicating the potential of ZnO NPs in drug delivery.^130^ Likewise, Abdel-Mageed *et al.*, developed a nano-hybrid system employing cyclodexterin-based NPs for the delivery of curcumin, the encapsulation efficiency of which was reported to be 90.2%.^131^ These findings correspond with our results and confirm the potential of Ag–ZnO NPs as a drug carrier.

#### Confirmation of diabetes induction

To confirm the induction of diabetes in diabetic model, the blood glucose levels (BGLs) of all groups were measured at the interval of 7 days for 3 weeks (Table 4). Following the administration of alloxan in positive control, rats exhibited a significant hypoglycemic state, with BGL elevating from 130.33 ± 7.50 mg dL^−1^ to 406.33 ± 9.55 mg dL^−1^, attributed to β-cell destruction and impaired insulin secretion.^132,133^ Persistent hyperglycemia is a driving factor behind oxidative stress, caused by overproduction of reactive oxygen species (ROS) and compromised antioxidant defense, which contributes towards lipid peroxidation and severe damage to hepatic and renal tissues.^134–137^ In close harmony to our findings, alloxan-induced diabetes contribute significantly in elevating hepatorenal injury markers, which indicates impaired hepatorenal function under hyperglycemic conditions.^138,139^

**Table 4:** Comparison of blood glucose levels across the groups over the trial period

Blood glucose level (mg dL^−1^)				
Groups	Day 1	Day 7	Day 14	Day 21
Control	131.33 ± 4.50	133.33 ± 3.05	132.67 ± 3.51	131.67 ± 3.51
Alloxan	130.33 ± 7.50	312.33 ± 6.10	383.0 ± 11.79	406.33 ± 9.55
Y1 (berberine)	125.67 ± 3.78	250.0 ± 7.55	190.67 ± 10.03	164.67 ± 7.23
Y2 (Ag–ZnO NPs)	122.33 ± 5.53	197.33 ± 3.51	163.33 ± 3.50	151.0 ± 5.87
Y3 (BBR-loaded Ag–ZnO NPs)	135.13 ± 4.39	158.0 ± 7.21	150.33 ± 8.50	145.33 ± 7.76

Following the treatment across all groups, a significant amelioration was observed in BGLs in Y3 (BBR–Ag–ZnONPs), from 158 ± 7.21 to 145.33 ± 7.76 mg dL^−1^, which indicates restoration of glucose metabolism as well as increased levels of insulin, suggesting restoration of β-cells, which is consistent with literature reporting enhanced anti-diabetic potential of nano-encapsulated BBR.^140–142^

#### Evaluation of liver function parameters

In CCl_4_-induced toxicity model, the classic hallmarks of acute hepatotoxicity were observed, including elevated levels of ALT, AST, ALP, and total bilirubin, and concomitantly reduced levels of albumin and total protein in serum.^50,143–145^ Furthermore, free radicals are rapidly generated during CCl_4_ metabolism *via* cytochrome P450 enzymes, including trichloromethyl and peroxyl radicals, which promote lipid peroxidation and contribute to cell necrosis.^146,147^ Comparatively, alloxan produces sustained hyperglycemia which further causes elevation in production of oxidants with concomitant reduced antioxidant activity, persistent oxidative stress and low-grade inflammation in hepatic cells, all contributing to liver injury.^148–152^ This suggests that while CCl_4_ induced a more pronounced and immediate spike in the levels of liver enzymes in present study, alloxan induced rather gradual metabolic impairment.

**Fig. 5:**
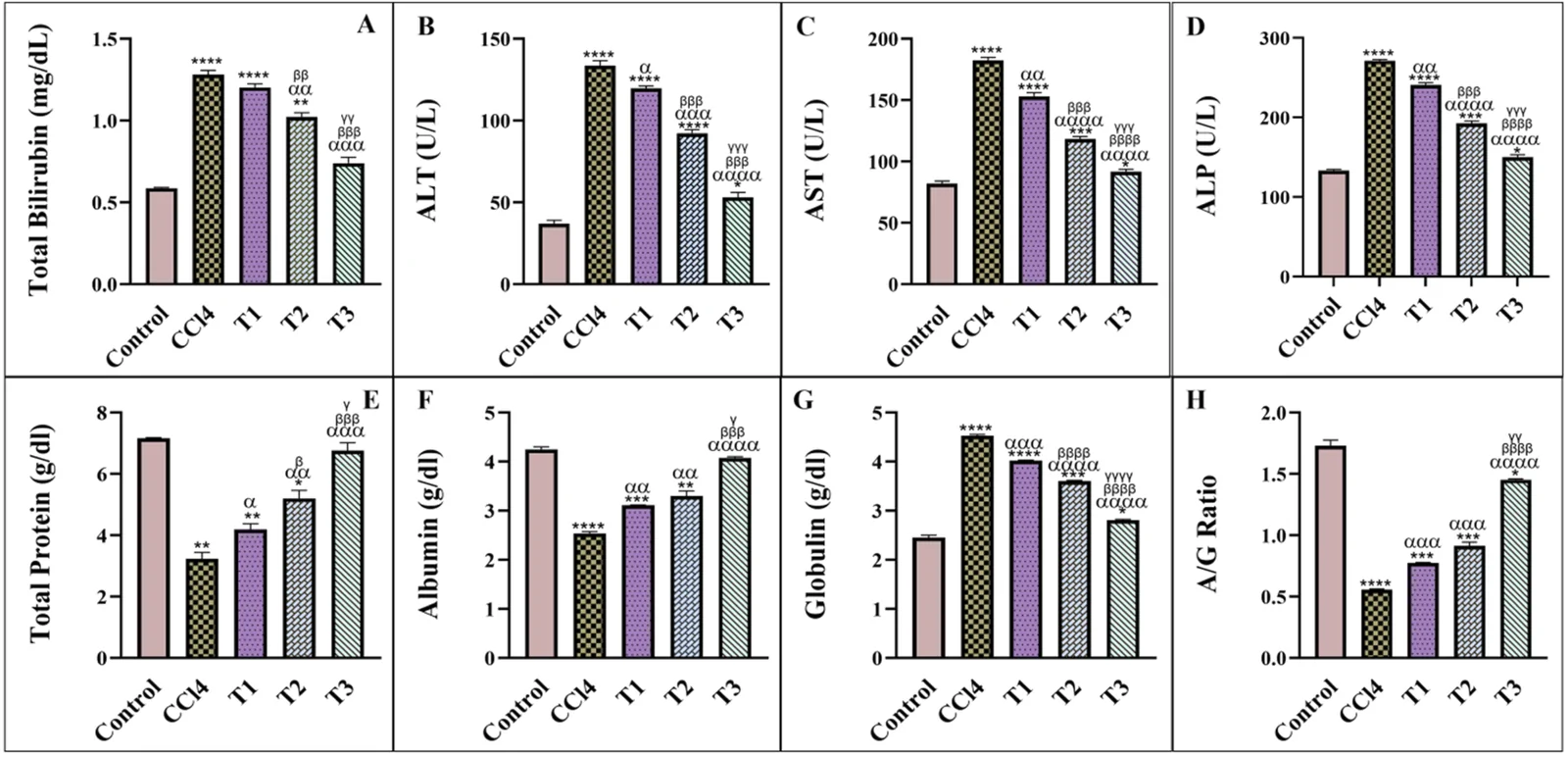
Hepatic biomarker in toxicity model. Serum levels of (A) total bilirubin, (B) alanine aminotransferase, (C) aspartate aminotransferase, (D) alkaline phosphatase, (E) total protein, (F) albumin, (G) globulin, and (H) A/G ratio in different experimental groups; T1 represent CCl_4_ + BBR treated group, T2 represent CCl_4_ + Ag–ZnO NPs treated group, and T3 represent CCl_4_ + BBR-loaded Ag–ZnO NPs treated group. Each column represent mean ± SD where *p* < 0.05, * represents significance between normal and other groups, α represents significance between diseased and other treatment groups, β represents difference between T1 and other treatment groups, and γ represents difference between T2 and T3. The number of stars *, **, ***, and ****, correspond to 0.05 (significant), 0.01 (more significant), 0.001 (highly significant), and 0.0001 (extremely significant), respectively. The number of α, correspond to significance from diseased group, α; 0.05 (significant), αα; 0.01 (more significant), ααα; 0.001 (highly significant), and αααα; 0.0001 (extremely significant). The number of β, correspond to significance from diseased group, β; 0.05 (significant), ββ; 0.01 (more significant), βββ; 0.001 (highly significant), and ββββ; 0.0001 (extremely significant). The number of γ, correspond to significance from diseased group, γ; 0.05 (significant), γγ; 0.01 (more significant), γγγ; 0.001 (highly significant), and γγγγ; 0.0001 (extremely significant).

**Fig. 6:**
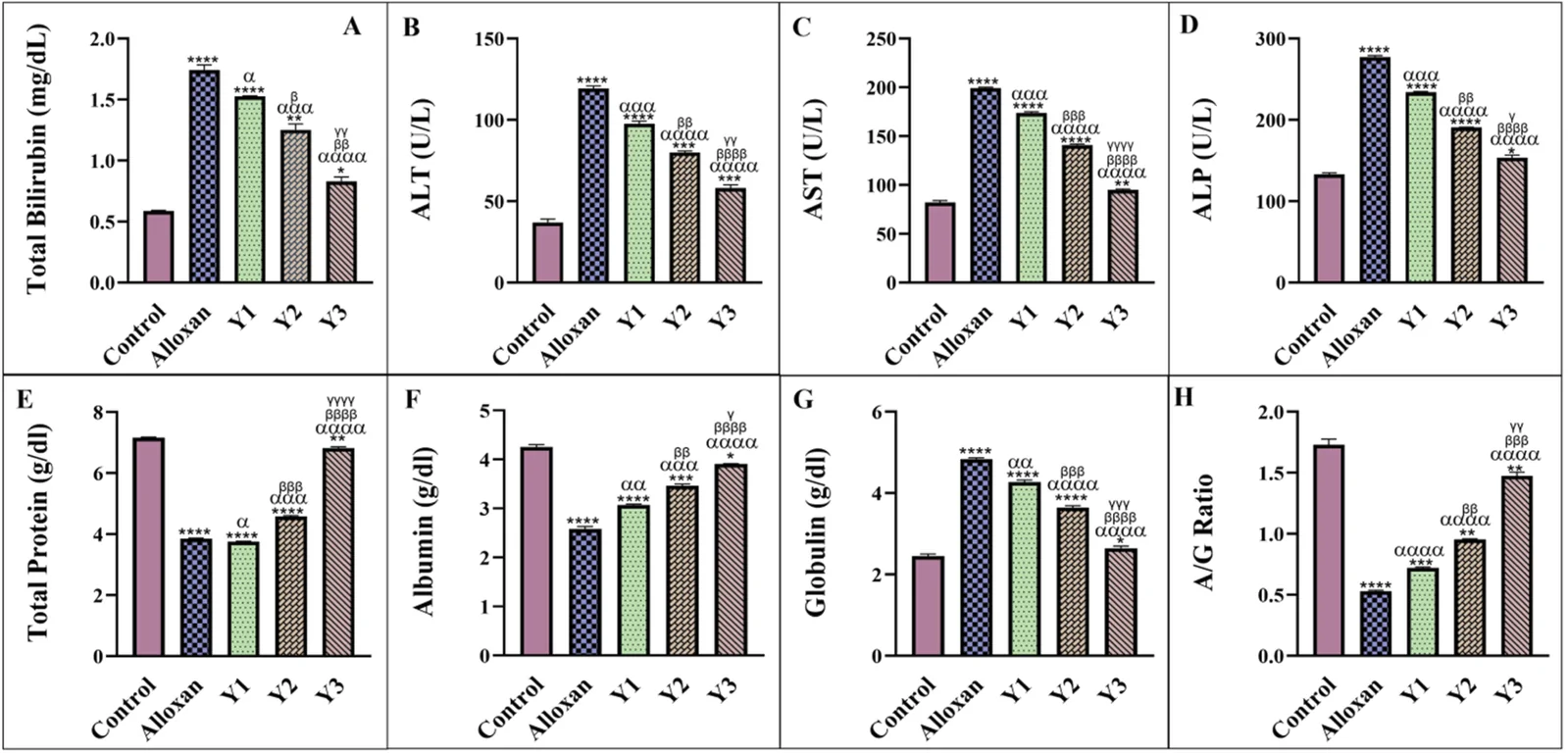
Hepatic biomarker in diabetic model. Serum levels of (A) total bilirubin, (B) alanine aminotransferase, (C) aspartate aminotransferase, (D) alkaline phosphatase, (E) total protein, (F) albumin, (G) globulin, and (H) A/G ratio in different experimental groups; Y1 represent alloxan + BBR treated group, Y2 represent alloxan + Ag–ZnO NPs treated group, and Y3 represent alloxan + BBR-loaded Ag–ZnO NPs treated group. Each column represent mean ± SD where *p* < 0.05, * represent significance between normal and other groups, α represent significance between diseased and other treatment groups, β represents difference between Y1 and other treatment groups, and γ represents difference between Y2 and Y3. The number of stars *, **, ***, and ****, correspond to 0.05 (significant), 0.01 (more significant), 0.001 (highly significant), and 0.0001 (extremely significant), respectively. The number of α, correspond to significance from diseased group, α; 0.05 (significant), αα; 0.01 (more significant), ααα; 0.001 (highly significant), and αααα; 0.0001 (extremely significant). The number of β, correspond to significance from diseased group, β; 0.05 (significant), ββ; 0.01 (more significant), βββ; 0.001 (highly significant), and ββββ; 0.0001 (extremely significant). The number of γ, correspond to significance from diseased group, γ; 0.05 (significant), γγ; 0.01 (more significant), γγγ; 0.001 (highly significant), and γγγγ; 0.0001 (extremely significant).

The biochemical analysis of liver enzymes demonstrates better improvement in CCl_4_ model as compared to diabetic model (Fig. 5 and 6), which may be associated with the antioxidant activities of BBR and NPs. Berberine has potent anti-inflammatory and anti-oxidant properties.^153–156^ In particular, BBR has been reported to mitigate the production of pro-inflammatory cytokines (TNF-α, IL-6, IL-8, and IL-1β) by inhibiting the activation of TLR4/MyD88/NF-κB pathway,^157–159^ while enhancing the activity of antioxidants (SOD, CAT, GSH) *via* the activation of AMPK/Nrf2 or AMPK/SIRT1 pathway.^160–162^ Likewise, the Ag–ZnO NPs have been reported to demonstrate anti-inflammatory and anti-oxidant activity, mitigating hepatic injury markers,^88,163–166^ which may be contributing to the enhanced hepatoprotective response of BBR-loaded Ag–ZnO NPs. Moreover, Saleh *et al.*, reported that BBR-loaded BSA NPs mitigated liver steatosis while improving liver function and autophagy signaling in toxicity models.^167^ Similarly, BBR-loaded PLGA NPs have also been reported to reduce ALT and AST levels in CCl_4_-induced toxicity,^168^ supporting the potential of NP-based delivery of BBR in enhancing its activity. Ag–ZnO NPs on the other hand, have been investigated as potent drug carriers that enhance anti-oxidant activity of drugs to improve the hepatic functions in toxicity models.^169–173^ To sum it up, the improvements observed in levels of liver function enzymes may reflect the combined antioxidant and hepatoprotective properties of Ag–ZnO NPs and BBR, resulting in greater attenuation of oxidative damage caused by CCl_4_ intoxication in hepatic cells.

Contrary to that, less pronounced results were demonstrated by diabetic model (Fig. 6), which may reflect the multifactorial nature of hepatic injury associate with diabetes, involving sustained metabolic overload, persistent oxidative and ER stress, immune activation, inflammation, lipotoxicity, and apoptosis.^134,174–177^ Bae *et al.*, reported that although anti-inflammatory and anti-oxidant therapies are promising in alleviating diabetes-induced hepatic damage yet the multifactorial nature of diabetes can limit the extent of therapeutic recovery.^178^ Although BBR has been reported to alleviate oxidative stress *via* the activation of AMPK and NF-κB pathway, and improve lipid/glucose metabolism, the underlying metabolic insult may persist by additional processes, including AGE accumulation, activation of PKC, over-activity of hexosamine pathway, polyol pathway flux, chronic inflammation, and compromised mitochondria.^179–184^ These findings suggest that anti-inflammatory and anti-oxidant therapies may be more effective against the oxidative component of CCl_4_-induced injury, whereas recovery from multi-factorial diabetic hepatic injury may be influence by additional metabolic and inflammatory disturbances.

#### Evaluation of kidney function parameters

**Fig. 7:**
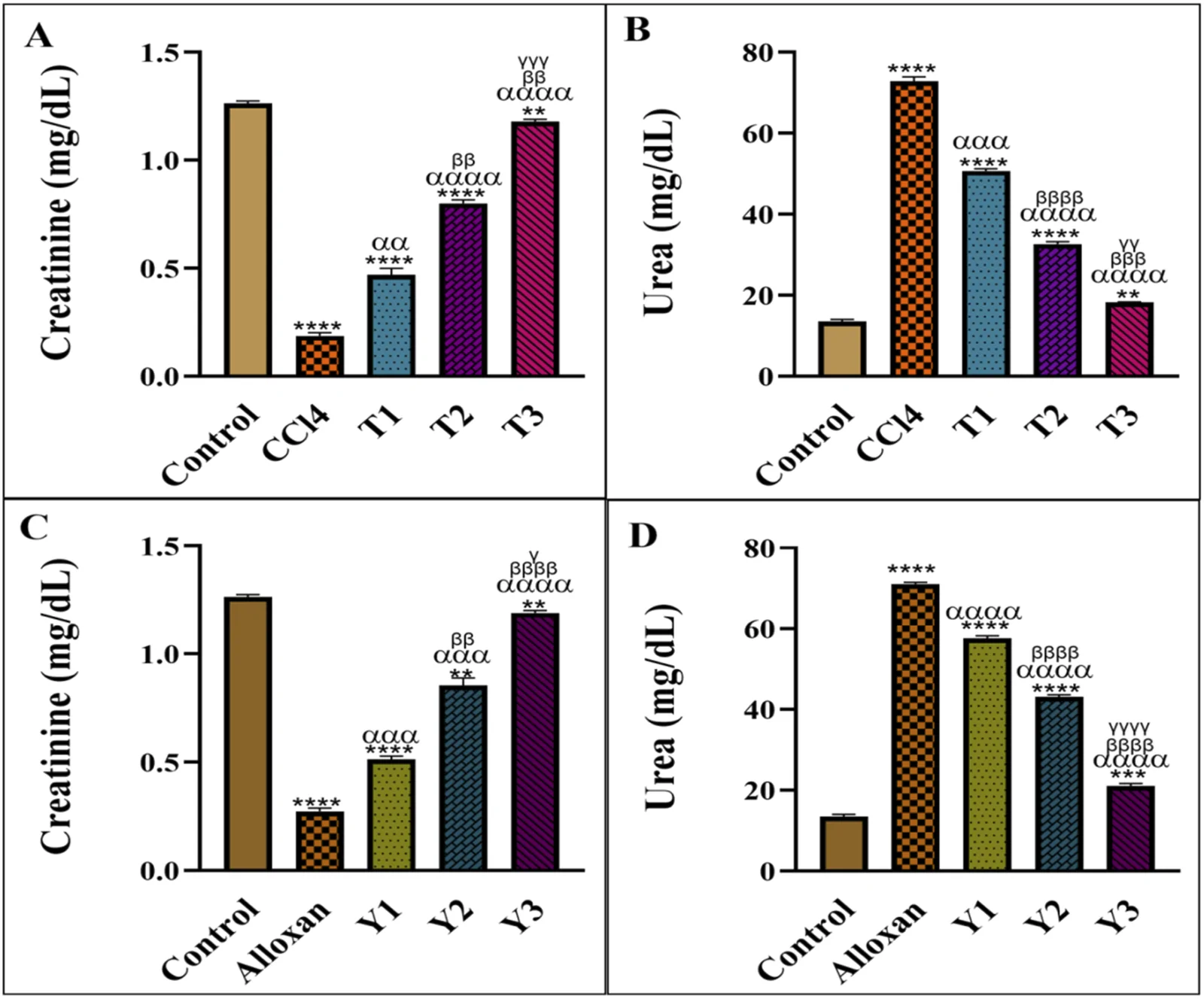
Renal biomarkers in toxicity (top) and diabetic (bottom) models. Serum level of (A) creatinine and (B) urea in different toxicity groups; T1 represent CCl_4_ + BBR treated group, T2 represent CCl_4_ + Ag–ZnO NPs treated group and T3 represent CCl_4_ + BBR-loaded Ag–ZnO NPs treated group, and serum level of (C) creatinine and (D) urea in diabetic groups; Y1 represent alloxan + BBR treated group, Y2 represent alloxan + Ag–ZnO NPs treated group, and Y3 represent alloxan + BBR-loaded Ag–ZnO NPs treated group. Each column represent mean ± SD where *p* < 0.05, * represent significance between normal and other groups, α represent significance between diseased and other treatment groups, β represents difference between T1/Y1 and other treatment groups, and γ represents difference between T2/Y2 and T3/Y3. The number of stars *, **, ***, and ****, correspond to 0.05 (significant), 0.01 (more significant), 0.001 (highly significant), and 0.0001 (extremely significant), respectively. The number of α, correspond to significance from diseased group, α; 0.05 (significant), αα; 0.01 (more significant), ααα; 0.001 (highly significant), and αααα; 0.0001 (extremely significant). The number of β, correspond to significance from diseased group, β; 0.05 (significant), ββ; 0.01 (more significant), βββ; 0.001 (highly significant), and ββββ; 0.0001 (extremely significant). The number of γ, correspond to significance from diseased group, γ; 0.05 (significant), γγ; 0.01 (more significant), γγγ; 0.001 (highly significant), and γγγγ; 0.0001 (extremely significant).

Renal stress was exhibited by both models, demonstrated by a spike in urea levels and reduction in creatinine levels, reflecting compromised renal clearance with concomitant enhanced catabolism of proteins under stress.^185–188^ Furthermore, creatinine is mainly synthesized in liver and kidney, a drop in creatinine levels may suggest compromised hepatic and renal function.^189^ Among the treatments, BBR-loaded Ag–ZnO NPs exhibited the best results, specifically in toxicity model, restoring the blood urea, whereas both models exhibited a similar degree of improvement in creatinine levels (Fig. 7). This reflects that as CCl_4_ causes a direct and intense oxidative stress leading to hepatorenal damage and inflammation, the antioxidant and anti-inflammatory activity of BBR-loaded Ag–ZnO NPs may provide greater protection against oxidative stress in this model as compared to persistent metabolic disturbances in diabetic model.^164,190–192^

#### Evaluation of lipid profile

**Fig. 8:**
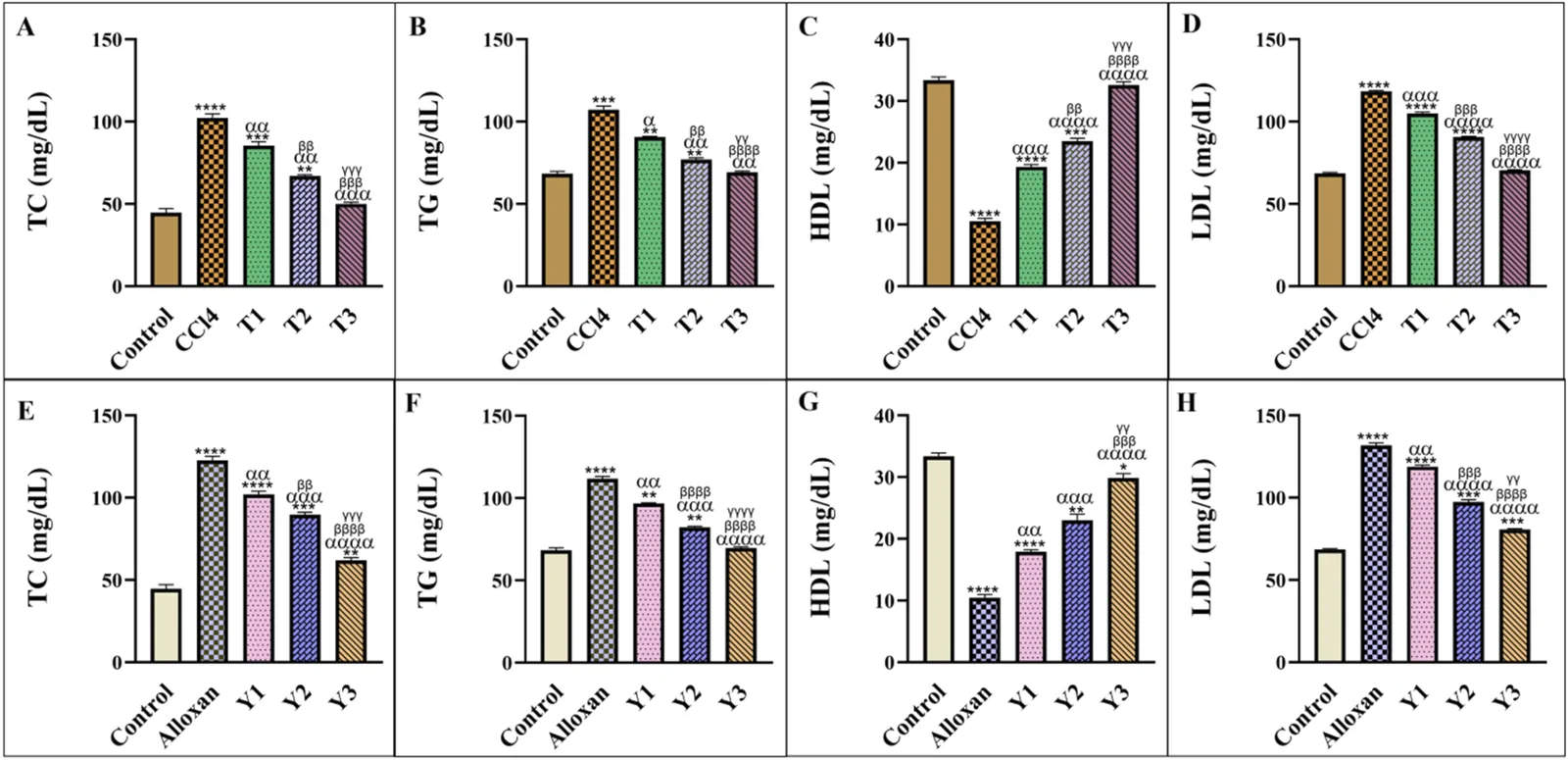
Lipid profile of toxicity (top) and diabetic (bottom) models. Expression of (A) TC, (B) TG, (C) HDL, and (D) LDL in different toxicity groups; T1 represent CCl_4_ + BBR treated group, T2 represent CCl_4_ + Ag–ZnO NPs treated group and T3 represent CCl_4_ + BBR-loaded Ag–ZnO NPs treated group. Expression of (E) TC, (F) TG, (G) HDL, and (H) LDL in different diabetic groups; Y1 represent alloxan + BBR treated group, Y2 represent alloxan + Ag–ZnO NPs treated group, and Y3 represent alloxan + BBR-loaded Ag–ZnO NPs treated group. Each column represent mean ± SD where *p* < 0.05, * represent significance between normal and other groups, α represent significance between diseased and other treatment groups, β represents difference between T1/Y1 and other treatment groups, and γ represents difference between T2/Y2 and T3/Y3. The number of stars *, **, ***, and ****, correspond to 0.05 (significant), 0.01 (more significant), 0.001 (highly significant), and 0.0001 (extremely significant), respectively. The number of α, correspond to significance from diseased group, α; 0.05 (significant), αα; 0.01 (more significant), ααα; 0.001 (highly significant), and αααα; 0.0001 (extremely significant). The number of β, correspond to significance from diseased group, β; 0.05 (significant), ββ; 0.01 (more significant), βββ; 0.001 (highly significant), and ββββ; 0.0001 (extremely significant). The number of γ, correspond to significance from diseased group, γ; 0.05 (significant), γγ; 0.01 (more significant), γγγ; 0.001 (highly significant), and γγγγ; 0.0001 (extremely significant).

Both models exhibited dyslipidemia following the induction of toxicity, reflecting a compromised lipid profile, with elevated levels of TC, TG, LDL and reduced HDL (Fig. 8). The BBR-loaded Ag–ZnO NPs were observed to improve dyslipidemia in both models, with significant improvement in CCl_4_-induced toxicity model, restoring the lipid profile close to control levels as compared to diabetic model. In CCl_4_-induced toxicity, hyperlipidemia may result from disturbances in lipid metabolism through lipid peroxidation, membrane damage, and hepatic damage.^146,147,193^ Various studies have reported that BBR activates the AMPK pathway, suppressing the expression of lipogenic genes, SREBP-1c and SCD1, which further mitigates the synthesis of triglycerides and improve lipid oxidation, which is consistent with the restored lipid profile in CCl_4_ model in our study.^162,194,195^ While in diabetic model, other than oxidative stress, persistent hyperglycemia causes lipogenesis along with the accumulation of lipid-derived signaling molecules, contributing to impaired fatty acid metabolism and diabetic dyslipidemia.^196,197^ This suggests that given the anti-oxidant potential of BBR and Ag–ZnO NPs, the nano-formulation may have been more effective in mitigating the CCl_4_-induced oxidative injury as compared to metabolic disturbances associated with diabetic model, thus resulting in restoration of the lipid profile in CCl_4_ model to a greater extent.

#### Evaluation of serum antioxidant profile

**Fig. 9:**
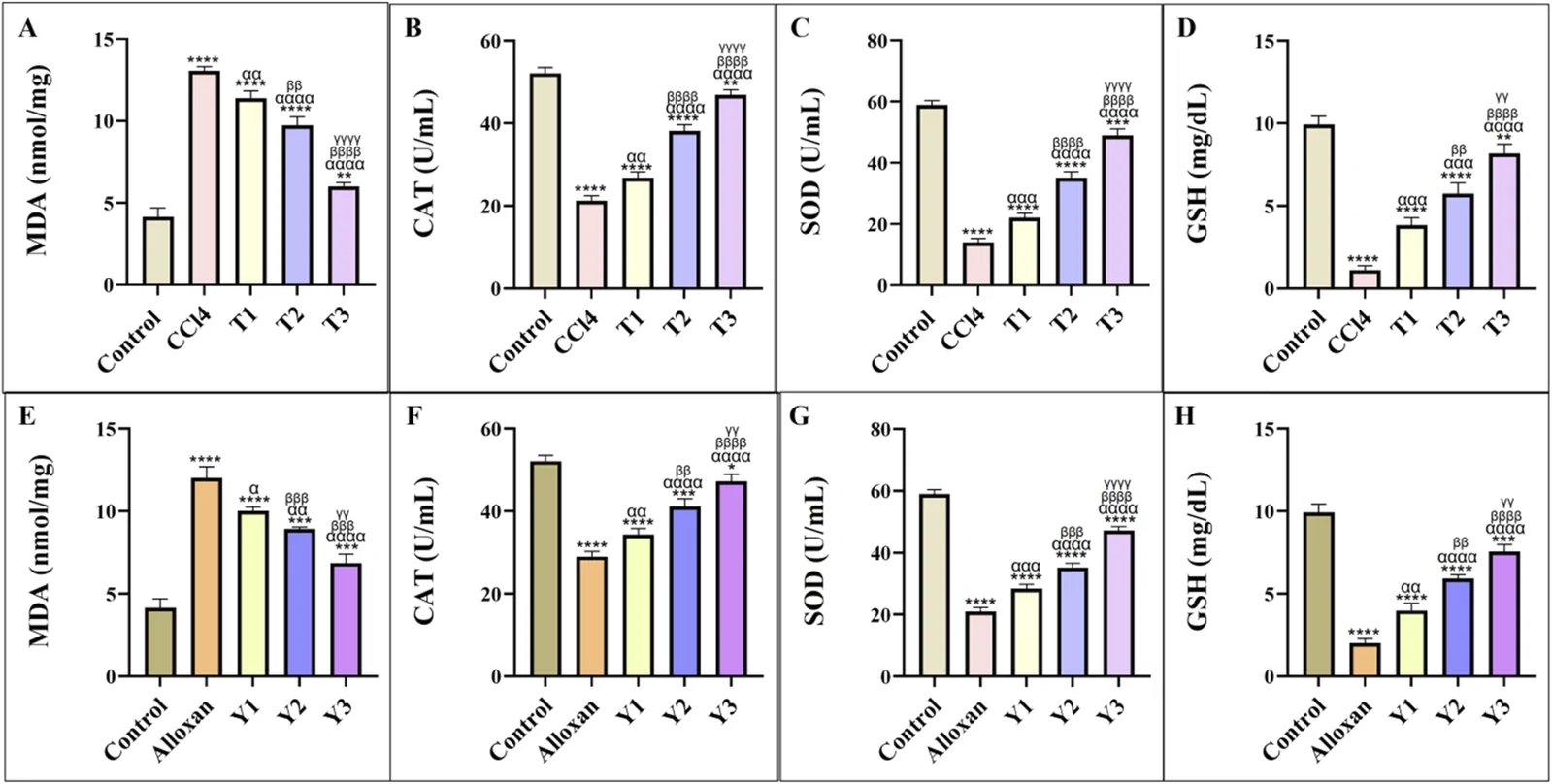
Evaluating the serum antioxiant profie in CCl_4_-induced toxicity model:(A) malondialdehyde (MDA), (B) catalase (CAT), (C) superoxide dismutase (SOD), and (D) reduced glutathione (GSH), T1 represent CCl_4_ + BBR treated group, T2 represent CCl_4_ + Ag–ZnO NPs treated group and T3 represent CCl_4_ + BBR-loaded Ag–ZnO NPs treated group. Evaluating the serum antioxiant profie in alloxan-induced toxicity model: (E) malondialdehyde (MDA), (F) catalase (CAT), (G) superoxide dismutase (SOD), and (H) reduced glutathione (GSH), Y1 represent alloxan + BBR treated group, Y2 represent alloxan + Ag–ZnO NPs treated group, and Y3 represent alloxan + BBR-loaded Ag–ZnO NPs treated group. Each column represent mean ± SD where *p* < 0.05, * represent significance between normal and other groups, α represent significance between diseased and other treatment groups, β represents difference between T1/Y1 and other treatment groups, and γ represents difference between T2/Y2 and T3/Y3. The number of stars *, **, ***, and ****, correspond to 0.05 (significant), 0.01 (more significant), 0.001 (highly significant), and 0.0001 (extremely significant), respectively. The number of α, correspond to significance from diseased group, α; 0.05 (significant), αα; 0.01 (more significant), ααα; 0.001 (highly significant), and αααα; 0.0001 (extremely significant). The number of β, correspond to significance from diseased group, β; 0.05 (significant), ββ; 0.01 (more significant), βββ; 0.001 (highly significant), and ββββ; 0.0001 (extremely significant). The number of γ, correspond to significance from diseased group, γ; 0.05 (significant), γγ; 0.01 (more significant), γγγ; 0.001 (highly significant), and γγγγ; 0.0001 (extremely significant).

To evaluate the oxidative status in both CCl_4_-and alloxan-induced toxicity models, serum MDA, CAT, SOD, and GSH were assessed (Fig. 9). Following the induction of toxicity in both models, the levels of serum MDA elevated significantly, while activity of CAT and SOD, and GSH levels decreased compared to control, indicating pronounced lipid peroxidation and disruption of serum redox-balance, which is consistent with literature.^198,199^ All the formulations exhibited different degrees of recovery in both models, however, BBR-loaded Ag–ZnO NPs exhibited the best results by decreasing the levels of MDA, and restoring the activity of SOD, CAT, and GSH levels. Their superior activity may be, in part, attributed to the synergistic effect of BBR and Ag–ZnO NPs. BBR has been reported to attenuate OS by activating various pathways including Nrf2/AMPK, P13K/Akt, and p38, that up-regulate the expression of SOD and HO-1.^200,201^ While Ag–ZnO NPs have demonstrated antioxidant activity by increasing the activity of SOD and CAT.^202,203^ Comparatively, T3 exhibited greater normalization of MDA, SOD, CAT, and GSH toward control values than Y3, probably because CCl_4_ exposure increases lipid peroxidation while suppressing endogenous antioxidant defenses, and effective antioxidant interventions can reverse these changes.^204,205^ However, apart from OS, alloxan-induced toxicity also results in β-cell injury and persistent hyperglycemia, which causes further metabolic disturbances that can sustain oxidative imbalance, which may account for these results.^206^

#### Histopathological analysis of liver and kidney

**Fig. 10:**
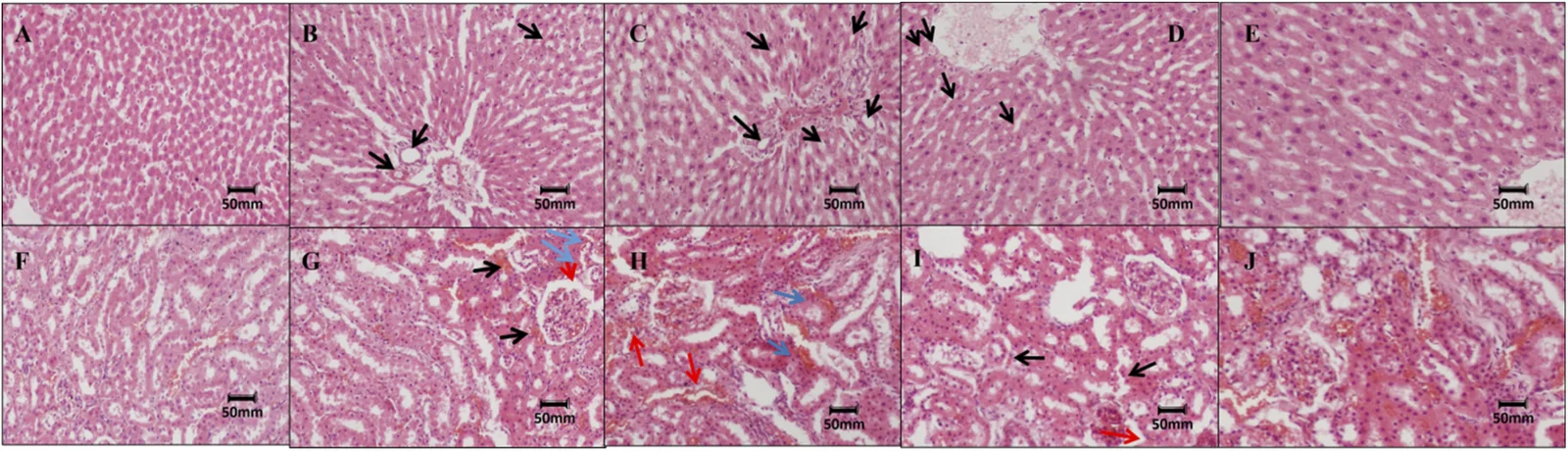
Histopathological evaluation of CCl_4_-induced toxicity, scale bar 50 nm. Hepatic: (A) control: normal architecture, (B) CCl_4_ (diseased): large vacoules indicating micro-vesicular fatty changes and swelling (arrow), (C) T1 (BBR): mild to moderate welling indicated by vacoules (arrow), (D) T2 (Ag–ZnO NPs): mild to moderate swelling with fewer hepatocytes being affected, (E) T3 (BBR-loaded Ag–ZnO NPs): minimal fatty degeneration, normal architecture of central vein and portal structures. Renal (F) control: normal architecture, (G) CCl_4_ (diseased): moderate to marked tubular epithelial swelling (blue arrow), noticeable peritubular congestion (black arrow), and cytoplasmic vascuolization (red arrow), (H) T1 (BBR): peritubular congestion showing RBCs in vessels (red arrow) and endothelial swelling (blue arrow), (I) T2 (Ag–ZnO NPs): mild swelling (black arrow) and degenerative changes (red arrow), (J) T3 (BBR-loaded Ag–ZnO NPs): pronounced renal restoration, mostly intact tubules, reduced swelling, and intact glomeruli and interstitium.

**Fig. 11:**
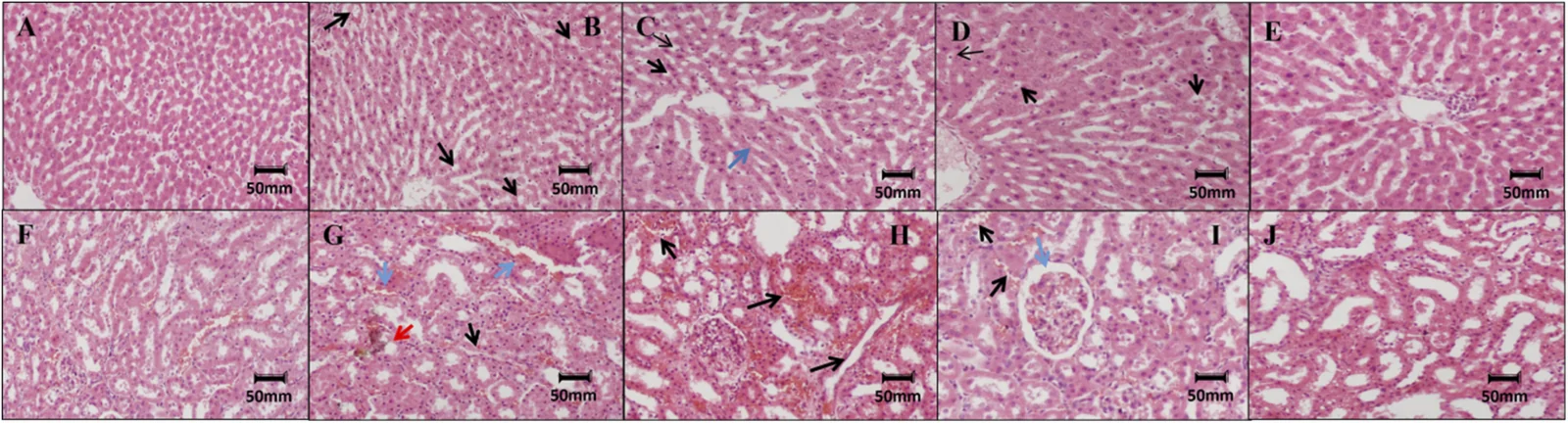
Histopathological evaluation of alloxan-induced toxicity, scale bar 50 nm. Hepatic: (A) control: normal architecture, (B) alloxan (diseased): large vacoules indicating micro-vesicular fatty changes and swelling (arrow), (C) T1 (BBR): mild to moderate swelling indicated by vacoules (arrow) and congestion (blue arrow), (D) T2 (Ag–ZnO NPs): mild vacuolation with fewer hepatocytes being affected, (E) T3 (BBR-loaded Ag–ZnO NPs): minimal fatty degeneration, normal architecture of central vein and portal structures. Renal (F) control: normal architecture, (G) alloxan (diseased): moderate to marked tubular degeneration (black arrow), noticeable peritubular congestion (blue arrow), and glomerulus shrinkage (red arrow), (H) T1 (BBR): peritubular congestion showing RBCs in vessels and tubular dilation (black arrow), (I) T2 (Ag–ZnO NPs): mild swelling (black arrow) and mild glomerulus shrinkage (blue arrow), (J) T3 (BBR-loaded Ag–ZnO NPs): normal glomerular, intact renal tubules, and minimal swelling.

In both models, the normal physiology of hepatic and renal structures was observed in control with intact central vein, portal area, and glomerulus (Fig. 10A, F and 11A, F). However, in CCl_4_ induced rats, severe hepatic and renal injury was observed, evident by micro-vesicular fatty change in many hepatocytes in hepatic cords and large vacuoles (Fig. 10B), indicating oxidative stress-induced steatosis, and peritubular congestion showing RBCs in vessels, atrophy, and endothelial swelling in kidney (Fig. 10G), which are the hallmarks of CCl_4_-induced toxicity.^207–211^ Partial hepatoprotection was shown by Berberine alone (T1), demonstrating fatty changes in some hepatic cords (Fig. 10C) and mitigation in swelling and preserved tubular structures, however mild degenerative changes were observed in both organs (Fig. 11H). Slightly better results were shown by Ag–ZnO NPs alone in preserving the hepatic architecture, with fewer hepatocytes being affected (Fig. 10D). However, BBR-loaded Ag–ZnO NPs showed pronounced results where hepatocytes were observed to be intact, minimal fatty degeneration, normal architecture of central vein and portal structures (Fig. 10E), along with kidneys showing mostly intact tubules, reduced swelling, and intact glomeruli and interstitium (Fig. 10F). BBR has been reported to significantly reduce CCl_4_-induced hepatic damage by modulating HMGB1/TLR4/NF-κB pathway, reducing inflammatory cell infiltration, and activating ferroptosis mechanism.^159,212,213^ BBR has also been reported to mitigate renal injury by mitigating the production of ROS, lipid peroxides, and down-regulating the expression of IL-6, TNF-α, NF-κB, Keap1/Nrf2, and AMPK/NF-κB pathways.^3,214–216^ ZnO and Ag NPs have been reported to exhibit hepatorenal protective activity, mainly through the mitigation of oxidative stress,^51,163,172,192,217,218^ though Ag NPs alone might cause toxicity, suggesting their co-administration with ZnO NPs.^34^ These findings suggest that BBR-loaded Ag–ZnO NPs may have utilized a number of pathways which altogether alleviated the oxidative stress, thereby restoring the hepatic damage which was mainly caused by oxidative stress.

In alloxan-induced toxicity, microvesicular fatty changes, vacuolization in cytoplasm, and structural damage were observed (Fig. 11B), which is consistent with studies indicating alloxan causes hepatic steatosis, attributed to insulin deficiency which triggers oxidative stress and disrupt hepatic metabolism.^219–221^ Likewise, the glomerular shrinkage, peritubular congestion, increased space of Bowman's capsule, and tubular vacuolization observed in kidney (Fig. 11G) indicates renal disruption.^151,222–224^ Studies have reported that persistent hyperglycemia up-regulates the expression of pro-fibrogenic TGF-β1 and pro-apoptotic p53, while disrupting the metalloproteinase, leading to renal injury.^225^ BBR partially restored the damage in both organs, characterized by somewhat restored hepatic cords, however, mild fatty changes were sustained, while peritubular congestion and swelling persisted in kidney. BBR has been reported to activate the AMPK signaling pathway, improving the antioxidant mechanism, while mitigating the triglycerides and regulating the lipid metabolism in diabetes, which may explain the partial organ recovery observed.^225–228^ Similar results were exhibited by Ag–ZnO NPs, demonstrating mild hepato- and nephroprotection as indicated by mitigation of fatty changes, better preservation of hepatocyte (Fig. 11D), less epithelial tubular changes and preserved structure of interstitium (Fig. 11I), which may be attributed to the metabolic regulating and anti-oxidative activities of NPs. As reported by Abd El-Baset *et al.*, administration of ZnO NPs in diabetic rats restored the function and structure of kidney, as indicated by the preserved structure of kidney, regulated levels of tubular markers like podocin, nephrin, and AQP11, and podocytes, along with mitigation in inflammation and fibrosis.^229^ Our results are in harmony with the study of Almori *et al.*, demonstrating that ZnO NPs restore the diabetic-induced damage in rats by improving the functions of kidney, like reduction of oxidative stress and inflammation, prevention of renal fibrosis and apoptosis, and delaying podocyte injury.^230^ The best results were exhibited by BBR-loaded Ag–ZnO NPs, restoring the architecture of both organs almost back to normal (Fig. 11E and J).

The same degree of recovery exhibited by both models, contrary to the different biochemical recovery, reflects that the experimental duration was not sufficient to produce overt structural damage, suggesting oxidative stress being the major driver of tissue injury.^231,232^ Thus, the progression of biochemical insult into irreversible histopathological injury can be prevented by early anti-oxidant therapy.^233^

#### Probable underlying mechanisms in alloxan and CCl_4_-induced toxicity

**Fig. 12:**
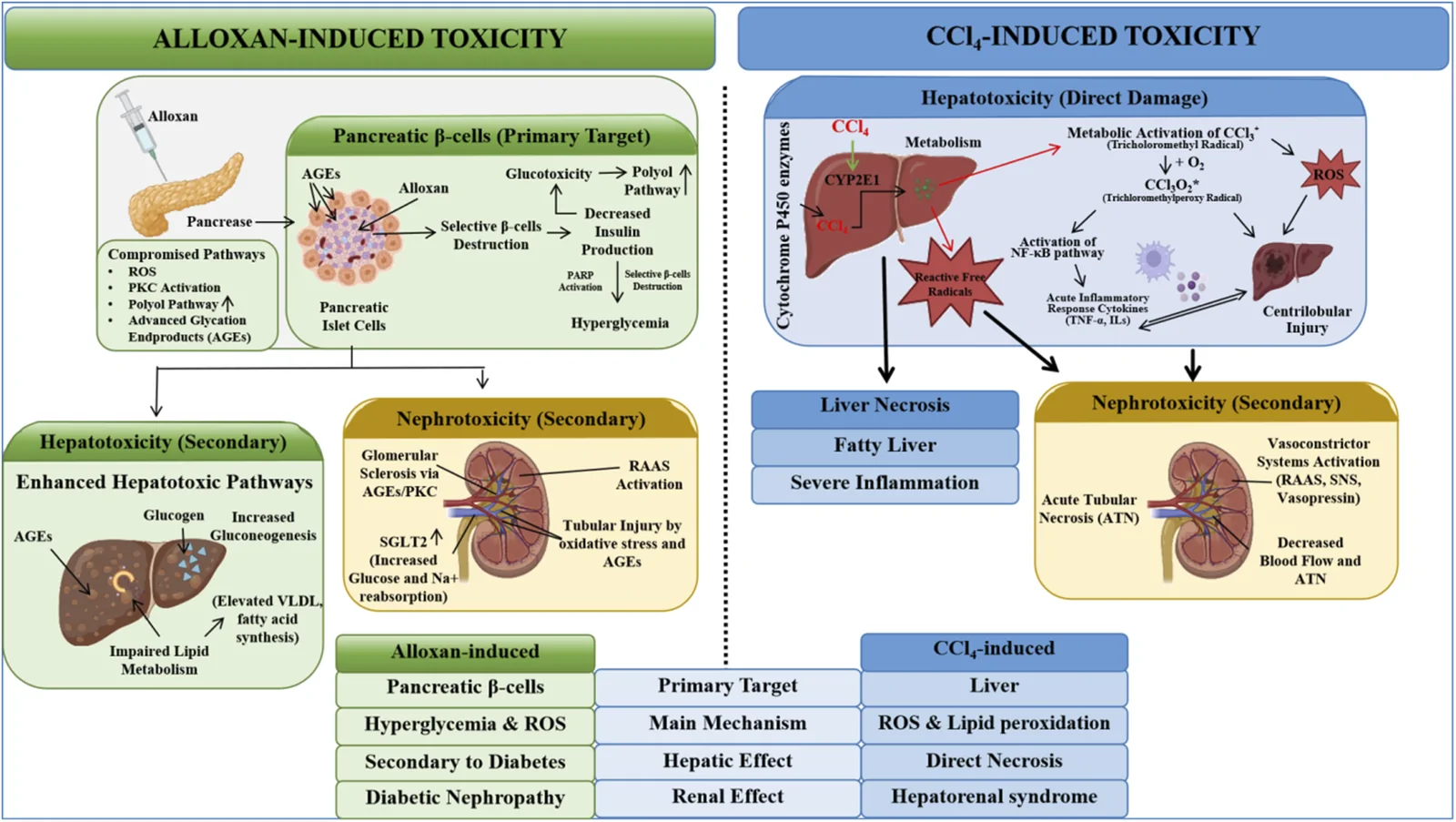
Comparative insight into the probable underlying mechanisms of CCl_4_-and alloxan-induced toxicity. Left: alloxan selectively destroys pancreatic β-cells, causing persistent hyperglycemia, which contributes to hepatorenal injury through AGEs accumulation, increased gluconeogenesis, RAAS (Renin Angiotensin Aldosterone System) activation, polyol pathway, and up-regulation of SGLT2 (sodium–glucose Cotransporter 2), altogether elevating the Very Low-Density Lipoprotein (VLDL) in liver and compromising the lipid metabolism. Right: CCl_4_ is metabolized in the liver by cytochrome P450 enzymes (CYPT2E1) into 
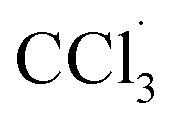
and 
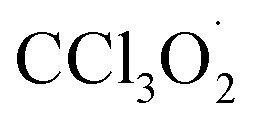
, triggering a cascade of pathways generating ROS (Reactive Oxygen Species), which damage both liver and kidneys, by activating RAAS and SNS (Sympathetic Nervous System).

Alloxan is one of the widely used diabetogenic agents, that selectively destroys pancreatic β-cells, elevating blood glucose levels.^234,235^ Alloxan undergoes cyclic autoxidation, generating hydroxyl radicals, superoxide, and hydrogen peroxide, which destroy β-cells, pertaining to their susceptibility to ROS-mediated injury and concomitant low expression of antioxidant enzymes.^236–238^ Moreover, activation of PARP (Poly(ADP-ribose) polymerase) triggered by alloxan-mediated DNA strand breaks depletes cellular NAD^+^ and ATP, leading to β-cells necrosis.^239–241^ Persistent hyperglycemia, owing to insulin deficiency, initiates the polyol pathway (glucotoxic), converting excess glucose to sorbitol, depleting the NADPH, impairing glutathione regeneration, and worsening the oxidative stress.^242–244^ Hyperglycemia facilitates the formation of Advanced Glycation End products (AGEs), which interact with RAGE receptors, activating a cascade of signaling pathways; MAPK/ERK, TGF-β, JNK, and NF-κB, causing severe inflammation and oxidative stress.^177,245–247^ These oxidative and metabolic disturbances can cause secondary damage to hepatic tissues *via* lipid peroxidation, compromised lipid metabolism, enhanced gluconeogenesis, and reduced storage of glycogen.^175,219^ Likewise, diabetes associated renal injury may involve AGEs accumulation and PKC-mediated glomerulosclerosis, concurrently, RAAS activation which further exacerbates the expression of sodium-glucose cotransporter 2 (SGLT2), leading to enhanced re-absorption of glucose, and oxidative stress-mediated tubular epithelial injury (Fig. 12).^248–250^

In CCl_4_ models, toxicity is attributed to the metabolism of CCl_4_*via* cytochrome P450 enzymes, which rapidly generate free radicals, including trichloromethyl 
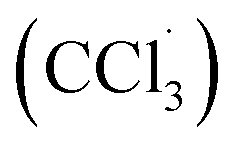
 and peroxyl radicals 
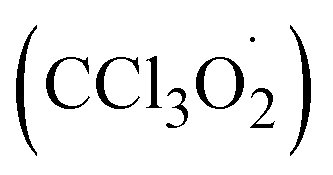
, triggering lipid peroxidation and hepatocyte necrosis.^146,251,252^ Additionally, NF-κB signaling pathway is activated by ROS, which up-regulates the expression of pro-inflammatory cytokines (IL-1β, IL-18, TNF-α, and IL-6), exacerbating inflammatory response and thus hepatic injury.^253–255^ Furthermore, compromised lipid metabolism and impaired β-oxidation may contribute to fatty liver disease *via* triglyceride accumulation.^256^ Similarly, the secondary renal damage may be attributed to oxidative stress from lipid accumulation, (Release of inflammatory mediators, and RAAS activation contributing to renal vasoconstriction.^219,257–259^ Renal functions are further impaired by ROS-mediated tubular-epithelial injury, which may further lead to acute tubular necrosis (ATN) (Fig. 12).^188,260,261^

#### Proposed protective mechanism of BBR-loaded Ag–ZnO NPs in combating toxicity

**Fig. 13:**
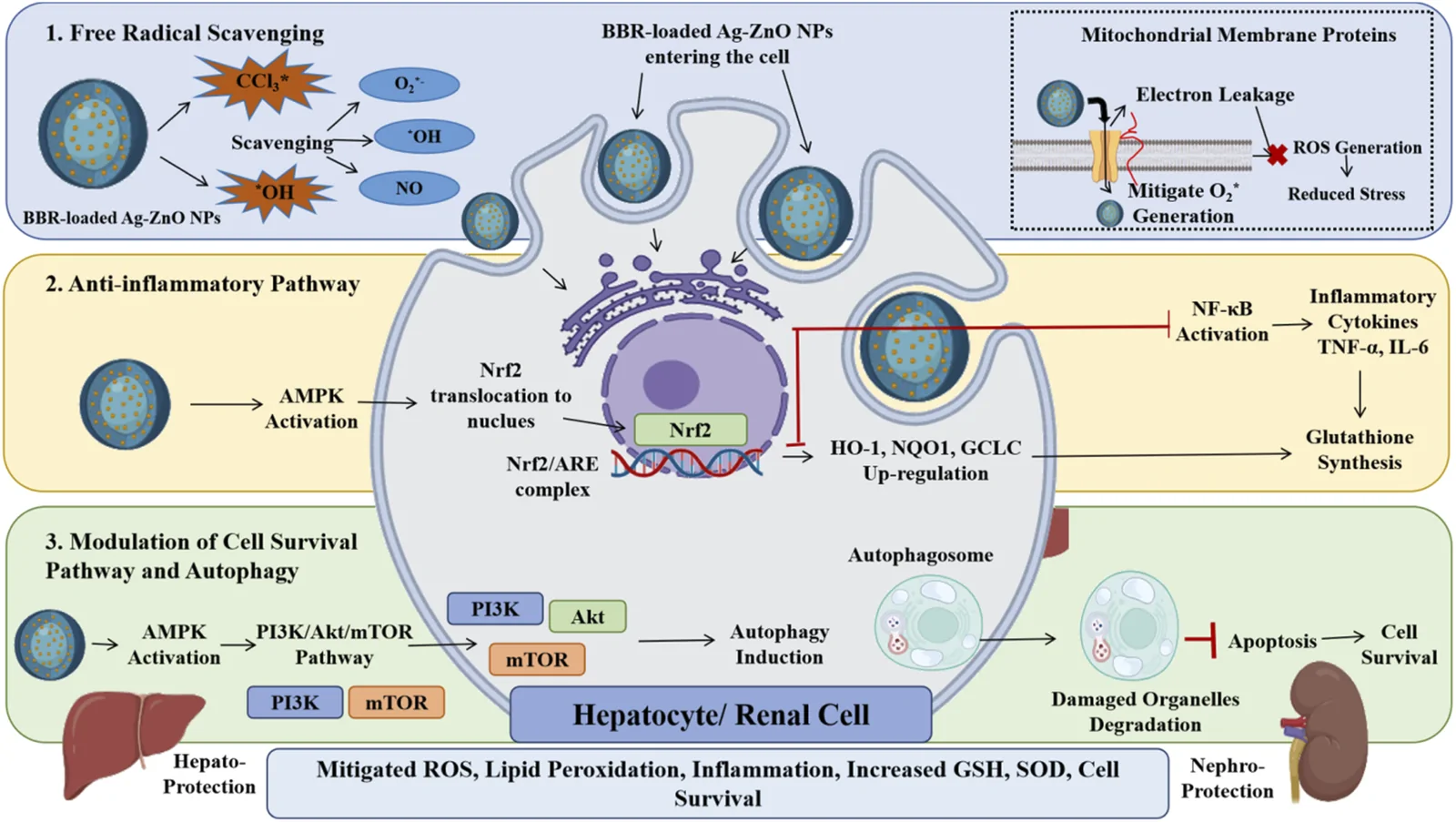
Proposed hepatorenal protective mechanism of BBR-loaded Ag–ZnO NPs against toxicity. (1) Free radical scavenging: BBR-loaded Ag–ZnO NPs neutralize the ROS, protecting the membrane damage, thus preventing the leakage of cellular enzymes. (2) Anti-inflammatory pathway: BBR-loaded Ag–ZnO NPs activate the AMPK pathway, enhancing the nucleus translocation of Nrf2, to upregulate the anti-inflammatory enzymes and inhinit inflammatory response *via* NF-κB. (3) Modulation of cell survival pathway and autophagy: AMPK activation leads to up-regulation of PI3K/Akt/mTOR pathway, which clears the damaged organelles to inhibits the inflammatory response. Altogether these mechanisms mitigate ROS (Reactive Oxygen Species), lipid peroxidation, inflammation, and increase GSH (Glutathione), SOD (Superoxide Dismutase), and anti-oxidant levels to restore homeostasis.

BBR and Ag–ZnO NPs have been reported to exhibit potent free radical scavenging activity and up-regulate anti-oxidant and anti-inflammatory signaling pathways, contributing to neutralization of ROS and alleviation of lipid peroxidation, protecting cellular membranes from oxidative insult and restrict enzyme leakage (Fig. 13(1)).^44,128,262,263^ BBR has been studied to activate the AMPK pathway, facilitating the translocation of Nrf2 to the nucleus, which may lead to activation of antioxidant response element (ARE) genes (CAT, SOD, HO-1, NQO1, GCLC), enhancing the endogenous anti-oxidant defense of cells (Fig. 13(2)).^156,226,264^ Similarly, both Ag and ZnO NPs have been reported to induce Nrf2-mediated anti-oxidant response *via* the activation of AMPK pathway.^265,266^ The suppression of NF-κB *via* AMPK activation, may further reduce the inflammatory response and thus cytokines-mediated tissue damage.^267^ AMPK-mediated autophagy through the modulation of mTOR, may inhibit inflammatory response by clearing out the damaged organelles, thereby restoring the homeostasis and functioning of hepatorenal tissues (Fig. 13(3)).^267–269^

## Conclusions

The present study demonstrates the hepatorenal protective potential of BBR-loaded Ag–ZnO NPs in mitigating oxidative stress and stress-induced hepatorenal damage. In both toxicity models, the respective toxicants compromised hepatorenal functions, elevated the levels of liver function (ALT, AST, total protein, bilirubin) and kidney function (urea, creatinine) enzymes, disrupted the lipid profile and endogenous antioxidant profile, and caused structural damage. BBR-loaded Ag–ZnO NPs were found to be more effective in mitigating the damage, by enhancing the anti-oxidant defense system, and restoring the levels of liver and kidney function enzmyes, specifically in CCl_4_-induced toxicity. The differential response observed between the experimental models, is attributed to the underlying mechanisms of injury. In alloxan-induced toxicity, persistent hyperglycemia and oxidative stress, continuously puts pressure on liver leading to secondary damage to liver and ultimately kidney. The nano-formulation effectively mitigated the oxidative stress and improved hepatic and renal function parameters, but the ongoing metabolic stress hindered complete normalization. Contrary to that, CCl_4_-induced toxicity, directly damaged hepatorenal tissues *via* the generation of ROS and lipid peroxidation, thus once the nano-formulation mitigated the oxidative insult, the hepatic and renal function parameters were more effectively normalized in this model. Altogether, these findings suggest that the effectiveness of BBR-loaded Ag–ZnO NPs is not model dependent but injury dependent, thus they exhibited greater effectiveness in combating direct oxidative toxicity compared to indirect (metabolic) toxicity, by inhibiting the source of oxidative insult.

Though BBR-loaded Ag–ZnO NPs demonstrated an excellent hepatorenal protective potential in this study, the underlying mechanisms, pharmacokinetic study, and toxicology study needs to be further explored to ensure the safety, optimal dosage, and long-term complications (if any) of nanoformulations prior to implementation in clinical settings.

## Author contributions

Javeria Shafique and Aleena Ali: methodology, investigation, visualization, and writing – original draft. Manal Nazim and Mubashra Inam: data analysis, data curation and methodology, and writing – review & editing. Mohamed Mohamed Soliman and Saed A. Althobaiti: resources, funding and writing – review & editing. Khalid S. Alotaibi: conceptualization and data visualization. Sumaira Anjum: conceptualization, supervision and writing – review & editing.

## Conflicts of interest

The authors declare that they have no known competing financial interests that could have appeared to influence the work in this paper.

## Data Availability

All data generated during this study are included in this article.
